# Lights and Shadows of Nutrient-Driven Keratinocyte Inflammation in Psoriasis

**DOI:** 10.3390/ijms262311652

**Published:** 2025-12-01

**Authors:** Desirèe Speranza, Alice Pantano, Chiara Cullotta, Giovanni Pallio, Mario Vaccaro, Michele Scuruchi, Natasha Irrera

**Affiliations:** 1 Department of Clinical and Experimental Medicine, University of Messina, Via C. Valeria, 98125 Messina, Italy; desiree.speranza@studenti.unime.it (D.S.); alice.pantano@unime.it (A.P.); michele.scuruchi@unime.it (M.S.); natasha.irrera@unime.it (N.I.); 2 Department of Biomedical and Dental Sciences and Morphological and Functional Imaging, University of Messina, Via C. Valeria, 98125 Messina, Italy; chiara.cullotta@studenti.unime.it (C.C.); giovanni.pallio@unime.it (G.P.)

**Keywords:** Psoriasis, keratinocyte, gut–skin axis, nutrient metabolism, oxidative stress

## Abstract

Psoriasis is a chronic inflammatory skin disease characterized by keratinocyte hyperproliferation, impaired differentiation, and dysregulated immune responses. Emerging evidence highlights the central role of keratinocytes as immune-competent cells that integrate signals from cytokines, metabolic cues, the gut–skin axis, and the tissue microenvironment. Key intracellular signaling pathways, including NF-κB, JAK/STAT, MAPK, and PI3K/AKT/mTOR, along with the IL-23/IL-17 axis, orchestrate keratinocyte-mediated inflammation and epidermal hyperplasia. Metabolic factors, nutrients, and redox balance further modulate these responses, while the intestinal microbiota and its metabolites, such as short-chain fatty acids, shape systemic and cutaneous inflammation. This review offers a critical, integrated perspective, that moves beyond descriptive summaries. We propose a conceptual framework in which the keratinocyte metabolic state, particularly the sirtuin/NAD+ axis, acts as a crucial convergence point for systemic nutritional, microbial, and inflammatory signals. Targeting sirtuins and associated pathways with natural or synthetic modulators represents a promising, host-centric strategy to restore keratinocyte function and reduce chronic inflammation. This synthesis underscores the potential of combining molecular, metabolic, microbial, and nutritional insights to develop personalized and effective approaches for psoriasis management.

## 1. Introduction

Psoriasis (PSO) is a chronic, autoimmune inflammatory skin disease of unclear cause, affecting about 2–3% of the global population [1]. Plaque PSO (PSO vulgaris), which accounts for more than 80% of cases [2], is the most common form of PSO, which manifests as raised and red patches covered with a silvery-white buildup of dead skin cells [3]. However, there are other less common and less frequent forms of PSO including guttate PSO, characterized by small dot-like lesions; inverse PSO, showing bright red lesions in skin folds; pustular PSO, marked by white pustules surrounded by red skin; erythrodermic PSO which occurs with itching and widespread red spots [4]. In addition, up to 30% of PSO patients develop psoriatic arthritis, characterized by joint pain and stiffness; nail involvement may also occur, often presenting as pitting and discoloration [5].

PSO typically occurs in individuals with a strong genetic predisposition and immune dysfunction, and is frequently associated with comorbid conditions such as obesity, diabetes, cardiovascular diseases, dyslipidemia, bowel diseases and psychiatric disorders, all associated with systemic inflammation [6,7].

Notably, nutrition appears to influence both the development and progression of PSO and its related comorbidities. Several studies report that patients with PSO often follow unbalanced diets, characterized by higher intakes of saturated fats and lower consumption of fibers and fish. Hence, such nutritional patterns may contribute both to the onset and severity of PSO [8].

Despite its low mortality rate, PSO is still considered a disabling condition for which there is no cure and that imposes a significant psychosocial, emotional, and economic burden on patients, primarily due to the visible and often stigmatizing nature of its symptoms.

While clinical assessment is the mainstay for diagnosing PSO, histopathological and immunohistochemical analyses of affected skin provide essential support in both diagnostic confirmation and understanding the disease pathogenesis [2]. While significant reviews have focused individually on the inflammatory pathways or the role of genetics in PSO, few have provided a mechanistically integrated synthesis linking the external environment (diet, nutrients, and the gut microbiota) directly to the intrinsic metabolic and signaling machinery of the keratinocyte (KC). The main objective of this review is to bridge this gap. We provide a focused analysis of how dietary components, and their resulting metabolites (e.g., short-chain fatty acids) modulate metabolic regulators, specifically sirtuins within KCs. By establishing this clear link, we aim to propose a novel therapeutic paradigm centered on metabolic and nutritional modulation as a strategy to dampen the KC’s inflammatory response, offering a non-immunosuppressive approach to management. To address this gap in knowledge, a structured literature search was conducted using the PubMed database. The search covered the last 20 years (2006–2025). The following search terms and combinations were used: “microbiota and psoriasis”; “nutrition and psoriasis”; “IL 17/23 axis and keratinocytes”; “NF-kb pathway and psoriasis”; “JAK-STAT pathway and psoriasis”; PI3K/Akt/mTOR pathway and psoriasis; “MAPK pathway and psoriasis”; “ROS and psoriasis”; “lipid peroxidation and psoriasis”; Sirtuin and psoriasis. Both preclinical and clinical studies were considered for inclusion. Studies were included if they: (i) were published in peer-reviewed journals between 2006 and 2025, (ii) reported experimental, translational, or clinical evidence relevant to nutrient-driven keratinocyte activation or inflammatory pathways in psoriasis, (iii) focused on human or validated in vitro/in vivo psoriasis models, (iv) were written in English. Studies were excluded if they: (i) were written in a language other than English, (ii) lacked clear information regarding patient characteristics (e.g., unspecified ethnicity, undefined diagnostic criteria), (iii) did not report the number of patients, (iv) were commentaries, conference abstracts, or non–peer-reviewed sources, (v) addressed psoriasis only indirectly or without specific reference to keratinocyte biology, nutrient-related pathways, or inflammatory signaling.

## 2. Role of Keratinocytes as Immune-Competent Skin Cells

The pathogenesis of PSO involves intricate crosstalk between KCs, immune cells, and other resident skin components. Initially, PSO was considered a predominantly immune-mediated disorder, as early studies identified cytotoxic T cells located near dermal and epidermal capillaries within psoriatic plaques. In fact, over the last two decades, PSO has been classified as a Th1-mediated disease due to the involvement of IFN-γ and TNF-α secreted by Th1 cells and IL-4, IL-5, and IL-13 released by Th2 cells [9]. Later, researchers highlighted the role of IFN-γ and IL-12 as key inflammatory drivers leading to the discovery of IL-23, a cytokine sharing the p40 subunit with IL-12 but containing a unique p19 subunit. By the early 2000s, IL-23 had been shown to induce IL-17A production by a distinct T cell subset, leading to the identification of Th17 cells and expanding the classical Th1/Th2 paradigm [10].

In the context of cytokine network dysregulation, where the IL-23/IL-17 axis plays a central pathogenic role, KCs were traditionally regarded as passive effectors of immune cell activity. However, they are now recognized as active immune drivers of both the onset and progression of PSO [11].

KCs serve as key sources of immune mediators. In pathological conditions, they exhibit abnormal and enhanced proliferative activity, leading to epidermal hyperplasia and impaired differentiation, which results in the accumulation of aberrantly matured KCs [12].

As integral components of the innate immune system, KCs respond to initial triggers and pro-inflammatory signals by releasing antimicrobial peptides, including S100 alarmins (S100A7, S100A8, S100A9), hBD2, and self-nucleotides [13], which promote the activation of plasmacytoid dendritic cells (pDCs). This, in turn, induces the maturation of myeloid dendritic cells (mDCs), which produce IFN-α, IFN-γ, TNF-α, IL-1β, and IL-23, thereby initiating psoriatic inflammation [14].

Moreover, stressed KCs produce a wide range of chemokines, including CXCL1/2/3, CXCL8, CXCL9/10/11, CCL2, and notably CCL20 [12]. The latter plays a key role in recruiting IL-17-producing CCR6^+^ Th17 cells and γδ T cells [15]. In addition, IL-17A stimulates KCs to express and secrete IL-25, which, through an autocrine loop, promotes a pro-inflammatory phenotype and enhances KCs hyperproliferation [16].

These mediators perpetuate the inflammatory response by attracting additional immune cells, such as neutrophils, DCs and macrophages [12].

Finally, KCs, together with fibroblasts and endothelial cells, contribute to tissue remodeling by promoting endothelial cell activation, proliferation, and extracellular matrix deposition [17]. The sustained crosstalk between KCs and immune cells, particularly Th17 cells, drives the development and chronicity of PSO, which is characterized by KC hyperproliferation, aberrant differentiation, vascular dilation and hyperplasia, as well as infiltration of inflammatory leukocytes [18,19].

## 3. The Gut–Skin Axis in Psoriasis: Short-Chain Fatty Acids Linking Intestinal Microbiota to Cutaneous Inflammation

**Figure 1:**
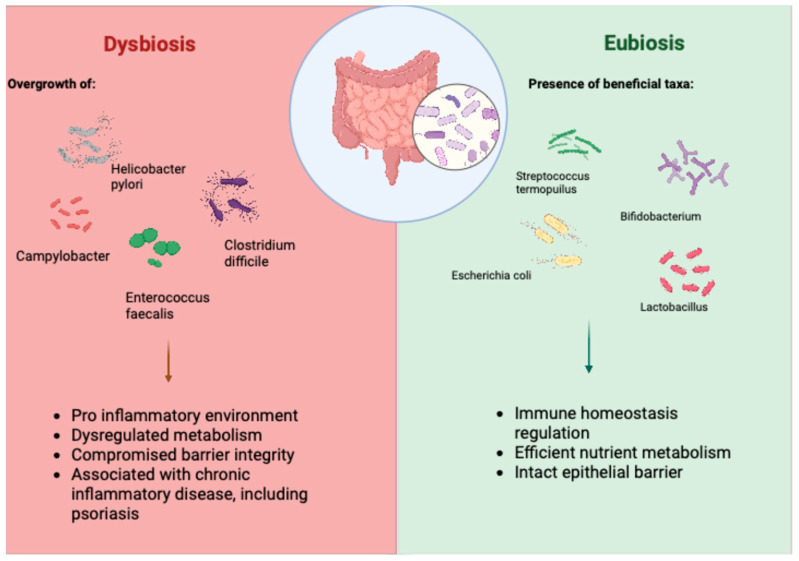
Gut eubiosis versus dysbiosis and their implications for intestinal and systemic homeostasis. Eubiosis is characterized by the presence of beneficial taxa such as Lactobacillus, Bifidobacterium, Streptococcus thermophilus, and commensal Escherichia coli, supporting efficient nutrient metabolism, maintenance of an intact epithelial barrier, and regulation of immune homeostasis. In contrast, dysbiosis features the overgrowth of pathobionts including Campylobacter, Enterococcus faecalis, Helicobacter pylori, and Clostridioides difficile, leading to a pro-inflammatory environment, disrupted metabolic functions, and impaired barrier integrity. These alterations contribute to systemic immune imbalance and are associated with chronic inflammatory conditions, including PSO.

Growing attention has been directed toward the gut–skin axis, particularly the influence of the gastrointestinal microbiota in several conditions, including PSO. The human body (especially the gut and the skin, but also the mouth, vagina, and airways) hosts rich and varied microbial communities [20]. The colon, which contains an estimated amount of 10^14^ bacteria, is the principal reservoir of these symbionts. The intestinal ecosystem comprises many bacterial phyla such as *Firmicutes*, *Bacteroidetes*, *Actinobacteria*, *Proteobacteria*, *Fusobacteria*, and *Verrucomicrobia* and includes viruses, fungi, protozoa, and archaea; together they form a symbiotic partnership with their host, and this relationship is shaped by age, genetics, diet, and broader environmental exposures. In addition to nutrient transformation, gut microbes generate bioactive metabolites, promote immune maturation and tolerance, and contribute to skin homeostasis. In this context, dysbiosis has been associated with gastrointestinal disorders and autoimmune, neurological, and neoplastic diseases [21,22,23] (Figure 1). Notably, gut dysbiosis can disrupt mucosal tolerance, adversely affecting skin health and being associated with PSO. In particular, intestinal dysbiosis can compromise mucosal tolerance, negatively affecting skin health and is associated with PSO. Therefore, immunoregulatory interaction along the gut–skin axis, which bidirectionally influences both microbial and host homeostasis, is central to maintaining systemic immune balance [24].

### 3.1. From Gut Microbiota Metabolites to Gut–Skin Axis

The main microbial metabolites that act along the gut–skin axis, exerting immunomodulatory effects on epithelial and hematopoietic compartments, include short chain fatty acids (SCFAs), trimethylamine (TMA)/trimethylamine N-oxide (TMAO), secondary bile acids and indoles derived from tryptophan [25]. For instance, elevated levels of TMAO have been found in PSO, linking altered microbial metabolism to both skin inflammation and cardiometabolic comorbidity. SCFAs, a family of ≤C6 carboxylates, formate (C1), acetate (C2), propionate (C3), butyrate (C4), and valerate (C5), are the dominant microbial metabolites in the colon. [25]. Acetate is the most abundant and acts as a systemic energy substrate that supports barrier integrity and modulates lipid metabolism and appetite [26,27]. Propionate acts primarily in the liver, reducing cholesterol synthesis, modulating gluconeogenesis, increasing IL-10, and limiting Th17 polarization [28]. Butyrate is the principal fuel for intestinal epithelial cells; it reinforces tight junctions (claudin/occludin), suppresses NF-κB signaling, enhances Treg function, and exerts additional epigenetic antineoplastic and neuroprotective effects [27,29].

SCFAs play a key role in immunity along the gut–skin axis through two main mechanisms: G-protein-coupled receptor (GPCR) activation and histone deacetylase (HDAC) inhibition. Among the SCFA-responsive receptors, GPR43 and GPR109A (HCAR2) are particularly relevant, as they are expressed in the colon and across multiple immune cell populations, where they mediate key metabolic and immunoregulatory effects [25]. Despite being commonly described as anti-inflammatory molecules, SCFAs can trigger opposite effects depending on the context. Acetate, for example, has been shown to promote inflammation through GPR43 activation. According to Seljeset et al., this interaction leads to increased production of IL-6 and chemokines such as CXCL1 and CXCL2 via activation of the TOR/PI3K/MAPK pathways [30]. Furthermore, Nadeem et al. demonstrated that oral acetate worsens inflammation by upregulating GPR43 expression in the epidermis and inducing IL-6 and dioxygenase-2 in an experimental model of IMQ-induced PSO [31]. However, acetate can also exert GPR43-independent anti-inflammatory effects: oral acetate attenuated inflammation and also suppressed LPS-induced TNF-α production in both Gpr43−/− mice and human monocytes [32]. In human PSO, reduced cutaneous expression of GPR43 and GPR109A has been observed; in these cases, topical application of butyrate restored receptor expression and shifted cytokine profiles towards an anti-inflammatory state (increased IL-10; decreased IL-17 and IL-6) [33]. Supporting its protective role, GPR109A deficiency exacerbated IMQ-induced skin inflammation, potentially due to impaired regulatory Treg function [34]. Overall, SCFAs promote Treg expansion and stability through coordinated receptor signaling and epigenetic regulation (Table 1) [25].

In addition to these receptor-mediated effects, SCFAs also engage in receptor-independent epigenetic regulation, primarily through HDACs inhibition.

HDACs are zinc-dependent enzymes that remodel chromatin structure and regulate gene expression programs essential for cell proliferation, differentiation, and inflammatory responses [35]. In PSO, HDAC1 mRNA is detectable in lesional skin and HDAC1 expression is upregulated alongside abnormal histone marks, implicating HDAC activity in the pathogenesis of the disease [36,37]. SCFAs enter the cytosol and nucleus and inhibit HDAC activity, which suppresses pro-inflammatory cytokines (e.g., TNF-α, IL-6) through downregulation of HDAC transcripts and attenuates systemic inflammation [38]. Moreover, in epidermal KCs, SCFA-mediated HDAC inhibition increases the expression of filaggrin and transglutaminase-1, promotes terminal differentiation, and supports the epidermal barrier. SCFAs also enhance DC morphology and antigen presentation through HDAC inhibition, influencing downstream T-cell responses [39,40]. DCs can induce the differentiation of Foxp3^+^ Tregs through the activation of GPR109A and GPR43 receptors [41]. The dysregulation of such processes in PSO favors the pathogenic response by tipping the equilibrium from Tregs toward effector cells and driving IL-17 responses. Kaisar et al. reported that SCFAs boost the activity of DCs by inhibiting HDACs and activating GPR109A, which drives T-cell activation [42]. SCFAs enhance retinoic acid production by gut DCs and support Foxp3 expression in Tregs, strengthening their anti-inflammatory activity [43,44]. Beyond shaping innate immunity, SCFAs also influence adaptive responses in psoriatic lesions, where Th1/Th17 cells dominate and Foxp3^+^ Tregs are reduced or dysfunctional. In this context, SCFAs help restore immune regulation by promoting pro-Treg signals (TGF-β1, retinoic acid) from DCs and macrophages [43,44]. These effects arise through GPR43/GPR109A signaling and epigenetic remodeling that increases histone acetylation [43,44]. Through these mechanisms, SCFAs recalibrate the Th17/Treg axis, suppress IFN-I-responsive CD8^+^ T-cell activity, and promote Treg expansion and function.

In addition, B lymphocytes, particularly regulatory B cells (Bregs or B10 cells), also play a role in modulating immune responses [43]. Bregs produce IL-10, which suppresses the IL-23/Th17 axis by promoting Treg induction and inhibiting Th17 differentiation. SCFAs support Breg development and enhance their regulatory function through mechanisms involving increased TCA cycle flux, p38-MAPK activation, HDAC inhibition, and glycolysis. As a result, SCFAs promote the expansion of IL-10–producing B10 cells, support polyclonal IgA and IgG secretion, and reduce apoptosis within this regulatory B cell population [44].

As reported, SCFAs attenuate key inflammatory circuits in PSO, thereby counteracting cytokine production, promoting immune cells repolarization and strengthening epithelial barriers. These converging signals lead to a broad attenuation of multiple pro-inflammatory pathways, including NF-κB, IL-23/IL-17, TNF-α/JAK–STAT, NLRP3 inflammasome and mTOR signaling [28,45,46].

**Figure 2:**
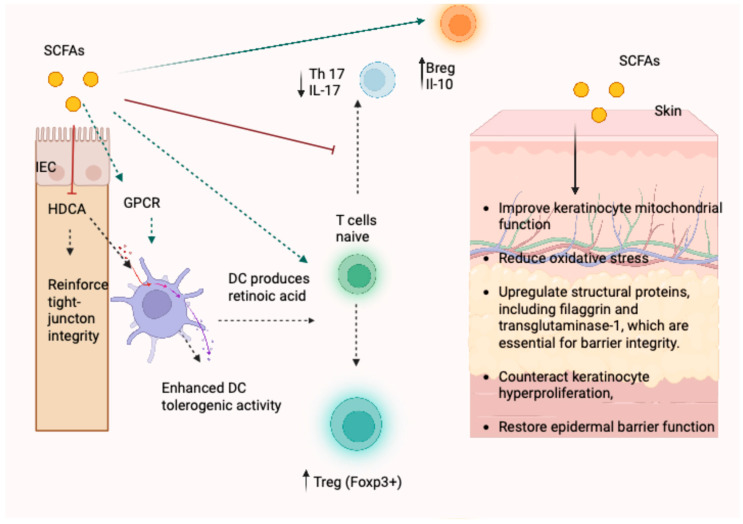
Mechanistic pathways through which SCFAs modulate the gut–skin axis in PSO. This schematic illustrates how the SCFAs butyrate and propionate modulate intestinal and skin immunity relevant to psoriasis. In the gut, SCFAs act on intestinal epithelial cells to reinforce tight-junction integrity and on dendritic cells to enhance tolerogenic activity and retinoic acid production. These signals, together with direct SCFA effects on naïve T cells, promote Foxp3^+^; regulatory T-cell differentiation and reduce Th17-associated IL-17 output. SCFAs also support regulatory B-cell IL-10 production. In the skin, SCFAs decrease oxidative stress, improve keratinocyte mitochondrial function, and upregulate structural proteins such as filaggrin and transglutaminase-1, thereby counteracting hyperproliferation and restoring epidermal barrier function.

PSO highlights the connection between a localized skin disease and systemic inflammation. Increasing evidence supports the role of the gut–skin axis in integrating dysbiosis, metabolic signaling, and immune modulation, factors linked to the intestinal microbiota that contribute to the pathophysiology of PSO [47,48]. The connection between gut dysbiosis and skin problems is striking and supports the concept of shared phylogenetic developmental origins and overlapping immune network (Figure 2). Two key elements in this relationship are the diversity of the microbiota, where eubiosis denotes immune tolerance, typically reflected by a low Firmicutes to Bacteroidetes ratio, and the integrity of the epithelial barrier. Damage to the gut barrier increases intestinal permeability, allowing the translocation of LPS, which activates Toll-like receptor 4 (TLR4) signaling and promotes KC hyperproliferation. SCFAs counteract IL-17–dependent inflammatory loops and contribute to restoring epithelial and immune balance [48,49]. By contrast, pro-inflammatory cytokines such as TNF-α, IL-23, IL-17A, IFN-α, and IFN-γ disrupt cytokine homeostasis [50]. Additionally, IL-17A and IL-22 secretion by DCs shaped by the microbiota in turn enhances KC hyperproliferation and neutrophil recruitment. Treg cells, in contrast, maintain immune tolerance via IL-10 production; however, dysbiosis weakens Treg function and amplifies inflammatory responses [45,51,52].

Simultaneously, LPS-induced NF-κB activation in KCs leads to increased production of IL-6 and IL-8 [53,54]. These multilayered interactions underscore the importance of preserving both microbiome balance and gut barrier integrity in PSO [46,55].

Importantly, the gut–skin axis is bidirectional: cutaneous inflammation can feed back to the gut through circulating cytokines (e.g., TNF-α, IL-17A, IL-22), antimicrobial peptides, and neuroendocrine stress signals [45]. These systemic mediators can alter gut permeability, mucus secretion, motility, and bile-acid signaling, thereby reshaping microbial composition [45,56]. This inflammatory feedback promotes dysbiosis, characterized by a loss of SCFA-producing taxa and an expansion of pathobionts, leading to reduced SCFA bioavailability and further loss of immune tolerance [45,56].

This skin-to-gut feedback intensifies systemic inflammation and creates a self-perpetuating loop with the gut-to-skin axis, reinforcing both the persistence and heterogeneity of the disease [51].

This schematic summarizes the key immunometabolic mechanisms by which the short-chain fatty acids butyrate and propionate influence intestinal and cutaneous immunity relevant to PSO. In the gut, SCFAs act on intestinal epithelial cells (IECs) through HDAC inhibition to reinforce tight-junction integrity and barrier function, thereby reducing permeability and LPS translocation. SCFAs also modulate dendritic cells (DCs) via GPR43/109A signaling and HDAC inhibition, promoting retinoic acid (RALDH) synthesis and enhancing their tolerogenic activity. These DC-derived signals, together with direct SCFA actions on naïve T cells, support the induction and stabilization of Foxp3^+^ regulatory T cells (Tregs), restoring the Treg/Th17 balance. SCFAs additionally promote regulatory B-cell (Breg) differentiation and IL-10 production, contributing to anti-inflammatory feedback loops. Systemically, increased Treg activity and reduced pro-inflammatory cytokine output mitigate pathogenic Th17 responses. In the skin, SCFAs reduce oxidative stress and enhance structural proteins such as filaggrin and transglutaminase-1, counteracting hyperproliferation and barrier disruption typical of psoriatic lesions. Together, these interconnected mechanisms illustrate how SCFA-dependent signaling integrates gut microbial metabolism with immune regulation and skin homeostasis, ultimately attenuating the chronic inflammatory circuits characteristic of PSO.

### 3.2. Link Between Gut Dysbiosis and Psoriasis: Evidence and Mechanisms

Within the emerging gut–skin axis framework, dysbiosis has been documented across inflammatory dermatoses and, specifically in PSO, is characterized by a loss of beneficial commensals and enrichment of pro-inflammatory taxa [57].

In a bidirectional two-sample Mendelian randomization (MR) analysis, Qian and colleagues used GWAS resources for the gut microbiome, covering 473 taxa at the species level, to investigate the causal relationship between microbial composition and PSO. In the forward direction, genetic instruments for microbial abundance identified 19 bacterial taxa with putative causal effects on PSO. The reverse analysis, which used genetic liability to PSO vulgaris as the exposure, implicated 13 microbial taxa as consequences of psoriatic disease [58]. Placed within the scope of existing literature, Qian’s findings align with reported compositional dysbiosis in PSO, including the relative abundance of *Prevotella* with concomitant reduction in Lachnospira and *Akkermansia muciniphila*, alongside low diversity in the sampled population. However, the trends for *Firmicutes*, *Actinobacteria*, and *Proteobacteria* across cohorts remain inconsistent [59]. Importantly, MR signals suggested a protective correlation with the family *Lentimicrobiaceae* along with certain taxa, including *Alistipes* and *Lactobacillus salivarius* [58]. This aligns with the immunomodulatory functions of SCFA-metabolizing microbes and protective bacteriocins that strengthen the microbial barrier [60,61]. Within the MR dataset, the species-level CAG-485 (sp002404675) and CAG-83 (sp000435555) were classified within the class *Clostridia*, a taxon linked to Treg induction and suppression of Th1/Th17-type inflammation [53]. On the other hand, numerous taxa were linked to a higher risk, such as the phylum *Omnitrophota*, the order *Flavobacteriales*, the families *Elusimicrobiaceae and Fusobacteriaceae*, CAGs 698 and 977, as well as genera *Bacillus AY*, *Brevibacillus B*, along with the species *Blautia* sp001304935 and *Desulfovibrio piger*. The mechanistic understanding of these associations is explained by the link of *Fusobacteriaceae* family members with mucosal inflammation as well as the hydrogen sulfide production by *Desulfovibrio piger* that may damage epithelium [54,62]. Finally, although MR suggested a protective effect for *Helicobacter* at the genus level [58], previous studies on *Helicobacter pylori* and PSO have shown contradictory results [63,64,65]. To translate these signals into actionable frameworks, Table 2 combines directionality, evidence grade, proposed mechanisms, and the hypothesized points of intervention, including dietary fiber and pre-, pro-, or synbiotics.

## 4. Nutrients, Metabolism, and Epigenetic Control of Keratinocyte Function in Psoriasis

Fatty acids, amino acids, glucose, and vitamins (especially A and D) influence KC activity and proliferation, which are central to the skin’s inflammatory response. Moreover, metabolic byproducts such as SCFAs, reactive oxygen species (ROS), and molecules like NAD+ and Sirtuins critically modulate inflammation and cellular function [75].

Building on this, growing evidence indicates that nutrigenomics and epigenetic modifications can shape KCs behavior, suggesting that dietary factors and specific supplements may regulate the expression of key PSO-related genes [76]. This integrative perspective highlights the promising role of nutraceuticals in PSO management, offering new insights on how nutrients and metabolic pathways converge to influence skin inflammation and KCs function [77].

In line with these molecular insights, clinical and lifestyle factors further emphasize the impact of nutrition and behavior on disease progression. Several risk factors, such as alcohol consumption, smoking, stress, sleep disturbances, sedentary behavior, diet, and intake of specific nutrients, have been identified as potential contributors to a more severe disease course [78].

PSO patients often show unbalanced dietary habits, characterized by a higher intake of saturated fats and simple sugars and a lower intake of components of the Mediterranean diet, such as fish, vegetables, and fibers. These imbalances are not coincidental but reflect a deep interaction between food and inflammatory processes [79].

Despite the recognized benefits of specific dietary patterns, adherence remains a clinical challenge. For example, the Mediterranean diet—although consistently associated with reduced inflammation and improved PSOseverity—is often difficult to maintain in the long term due to behavioral, cultural, or socioeconomic factors. Similarly, more restrictive approaches such as the ketogenic diet may show short-term metabolic advantages but frequently present adherence issues related to dietary monotony, gastrointestinal symptoms, and the need for sustained carbohydrate restriction. Such considerations underline that the potential impact of diet on PSO is deeply influenced not only by biological effects but also by the patient’s long-term ability to follow specific nutritional regimens [8,80,81].

Conversely, a balanced diet and regular physical activity may positively affect the condition of psoriatic patients. By providing an adequate quantity of polyunsaturated fatty acids, fibers (supplemented with prebiotics), and vitamin D, a healthy lifestyle can effectively modulate inflammation and improve the overall well-being of patients [82].

In summary, chronic inflammation and oxidative stress are key drivers of PSO, and diet can play a significant role in modulating these factors. Obesity is a considerable risk factor that worsens the disease and the response to treatment. Accordingly, dietary strategies such as a low-calorie diet, a gluten-free diet (for patients with gluten sensitivity), and particularly the Mediterranean diet are considered promising approaches to improving therapeutic outcomes [78].

### 4.1. Impact of Fatty Acids

Fatty acids are key modulators of immune function and KCs behavior. SFAs, predominantly found in processed and animal-derived foods, are associated with increased inflammation. They activate dendritic cells, triggering the release of pro-inflammatory cytokines like IL-1β and TNF-α, and can negatively affect regulatory T-cell function. In contrast, polyunsaturated fatty acids (PUFAs), particularly omega-3s fatty acids (n-3 FAs), like EPA and DHA, and omega-6s fatty acids (n-6 Fas), exert anti-inflammatory effects. They can decrease IL-6 levels, reduce TNF-α production, and inhibit the activation of the NLRP3 inflammasome, thereby helping to mitigate the inflammatory response [83].

Maintaining a balanced omega-6 and omega-3 ratio is crucial for establishing a healthy metabolic environment; the n-6/n-3 ratio ideally should not exceed 4:1, whereas typical Western diets often reach 10:1 or higher, promoting chronic inflammation [75]. This balance represents one aspect of dietary modulation of inflammation, which can be complemented by other nutrients such as amino acids and vitamins, each contributing to KC function and immune regulation [19].

Although some supplements and specific polyphenols have shown promising results in preliminary studies, a lack of large-scale, high-quality research currently limits clinical recommendations.

Oral supplementation studies have produced inconsistent results, often due to insufficient doses or uncontrolled dietary fat intake. In contrast, intravenous applications have shown more promising outcomes, leading to rapid clinical and immunological improvements. Fish oil supplementation also appears more effective when combined with other therapies, such as UVB, tacalcitol and etretinate. However, due to the lack of conclusive evidence, the National Psoriasis Foundation’s Medical Board does not recommend oral or intravenous fish oil supplementation in its dietary guidelines [84].

Beyond lipids, protein intake also plays a significant role in modulating inflammation. Diets rich in protein, particularly from red and processed meats, are associated with elevated levels of inflammatory markers, including C-reactive protein (CRP), IL-1, IL-6, and TNF-α. In contrast, plant-based or dairy proteins appear to have a more favorable effect on the metabolic environment. Some evidence even suggests that low-protein diets might reduce CRP levels more effectively, although the overall effects of protein intake remain conflicting [83]. Despite encouraging findings, studies investigating fatty acids and protein intake in PSO are generally limited by small sample sizes, heterogeneous study designs, lack of standardized supplementation protocols, and short follow-up periods. Moreover, dietary intake is often not strictly controlled, making it difficult to attribute clinical improvements solely to the interventions.

### 4.2. Impact of Carbohydrates

The impact of carbohydrates on PSO largely depends on their quantity, quality, and dietary source. Diets with a high glycemic load (GL) or glycemic index (GI) are associated with elevated levels of inflammatory markers, including CRP, IL-6, and TNF-α. In contrast, dietary fiber, a complex carbohydrate, exhibits anti-inflammatory effects. It is fermented by gut microbiota into SCFAs, which can significantly reduce CRP levels and contribute to a healthier metabolic environment [85]. Emerging evidence also suggests that very low-calorie ketogenic diets (VLCKDs) may inhibit inflammation by promoting the production of ketone bodies, like β-hydroxybutyrate. These molecules appear to block the NLRP3 inflammasome activation and improve oxidative stress, leading to a notable reduction in body weight and Psoriasis Area and Severity Index (PASI) scores in some patients [86].

Carbohydrates are classified as macronutrients and are important modulators of immune and inflammatory responses. High carbohydrate intake, particularly those associated with a high GL diet, is strongly correlated with high plasma concentrations of high-sensitivity C-reactive protein (hsCRP) in healthy women [87]. Studies conducted on elderly subjects have shown that a high GI or high GL diet led, after one year of follow-up, to an increase in IL-6 and TNF-α levels, as well as a concomitant decrease in leptin and adiponectin levels [87]. A hyperglycemic diet (containing 59–67% of Total Caloric Value, TCV) resulted in higher CRP concentrations. In contrast, a hypoglycemic diet (10–13% of TCV) led to decreased IL-6 levels [88]. The importance of carbohydrate quality was highlighted in a study in which a low-GL diet reduced CRP compared to a high-GL diet in participants, even though the diets were isocaloric (same calorie content) [89]. It should be noted that some reports suggest that the reductions in CRP and serum amyloid A (SAA) may be attributable to weight loss and not specifically to the carbohydrate content of the diet itself [90].

The term “fibre” is used to denote “complex carbohydrates”. Fiber, which is composed primarily of complex carbohydrates, serves as a prime example that underscores the significance of the source of carbohydrates and exerts a predominantly anti-inflammatory effect. The ingestion of a diet containing 30 g of fiber per day resulted in a substantial decrease in the levels of the inflammatory marker hsCRP [91].

A study of overweight and obese individuals revealed that supplementation with rice bran and rice husk powder, as part of a reduced-energy diet, led to a decrease in hsCRP and IL-6 [91]. Fiber has been demonstrated to retard the absorption of carbohydrates and impede the absorption of dietary lipids [92]. Some indigestible fibers are fermented in the colon to produce SCFAs [93,94].

VLCKDs represent an approach that aims to significantly restrict carbohydrates in order to induce physiological ketosis; the main feature of the VLCKD is a very low carbohydrate intake, typically less than 30–50 g/day (13–25% of total calories), with adequate protein intake and a total calorie intake of less than 800 kcal/day [95]. This restriction leads to an increase in the production of ketone bodies by the liver, which act as an alternative energy source and are the main mechanism responsible for the anorexigenic effect (reduction in hunger) [96].

The VLCKD is an effective strategy for managing overweight and obesity, associated with rapid and consistent weight loss (on average −10.0 kg to −15.6 kg depending on the duration of the ketogenic phase) [92]. The anti-inflammatory role of the ketogenic diet is supported by several pieces of evidence, such as the activation of the peroxisome proliferator-activated receptor gamma (PPAR-γ) and the hydroxycarboxylic acid receptor 2 (HCA2) and the inhibition of the inflammasome by beta-hydroxybutyrate, with a dose-dependent reduction in IL-1β and IL-18 [97]. Large-scale randomized prospective studies will therefore be necessary to systematically evaluate the sustainability, efficacy, and potential risks of VLCKDs in order to define its safer, evidence-based clinical use.

Studies on VLCKDs have shown a significant reduction in inflammatory and metabolic markers, including HbA1c, total cholesterol, and triglycerides. The VLCKD has shown promising results in the management of inflammatory diseases such as PSO, leading to a significant reduction in PASI scores and decreased levels of IL-1β and IL-2 [98].

In summary, while high glycemic load carbohydrates are associated with increased inflammation, complex carbohydrates (such as fiber) exert anti-inflammatory effects. Dietary regimens that drastically reduce carbohydrate intake, such as the VLCKD, exploit the metabolic mechanisms of ketosis to induce rapid weight loss and positively modulate the inflammatory state [92]. Overall, clinical trials investigating fatty acids and protein intake in PSO are constrained by important methodological weaknesses. Most available studies include relatively few participants, apply diverse supplementation regimens, and differ substantially in duration and outcome assessment. In many cases, dietary intake is not rigorously monitored, and co-interventions are inconsistently controlled, making it difficult to attribute observed changes specifically to the nutrient under investigation. These issues collectively limit the comparability of results and reduce the strength of conclusions that can be drawn from current evidence. Future studies will need to clarify the pathophysiological mechanisms underlying the observed benefits and identify which patient subgroups may benefit most from such dietary approaches.

### 4.3. Impact of Vitamins

Both Vitamin A and Vitamin D play a role in immune regulation, which is crucial for managing PSO. Retinoic acid, the active metabolite of Vitamin A, supports immune cell function, and its deficiency can impair the immune response. Vitamin D, on the other hand, has a more clearly defined role. PSO patients often exhibit low Vitamin D levels, which can disrupt immune balance [99].

The active form of Vitamin D, calcitriol, inhibits KC proliferation and promotes differentiation, making topical Vitamin D therapy an effective treatment [100].

However, the efficacy of oral Vitamin D supplementation remains inconsistent. Consequently, the National Psoriasis Foundation’s Medical Board recommends oral supplementation for patients with confirmed deficiency, primarily to prevent comorbidities, rather than for patients with normal serum levels [86].

Overall, nutrition plays a pivotal role in the development and progression of PSO and its comorbidities. Extensive evidence suggests that specific nutrients and dietary patterns can either exacerbate or ameliorate the disease. Therefore, a careful assessment of the patient’s diet and nutritional status, conducted by a dermatologist in collaboration with a nutritionist, can help guide personalized dietary strategies that complement conventional therapies [78]. Although nutritional interventions, such as vitamin D supplementation, show promising therapeutic potential in PSO, current evidence remains limited. This limitation is linked to individual variability due to genetic, environmental, and lifestyle factors, which further affects the robustness of evidence. In general, large-scale, randomized, controlled clinical trials are needed to rigorously validate the efficacy and safety of such nutritional strategies in the management of PSO [78,86,99].

Another emerging and promising field for understanding the complex mechanisms of PSO and its comorbidity, psoriatic arthritis, linked to nutrition, is metabolomics. This field involves the systematic analysis of metabolites, providing a functional snapshot of biochemical processes. Unlike other “omics,” metabolomics reflects the intricate interplay between genetics, environment (including diet), and cellular activity [101].

Advanced analytical techniques, such as mass spectrometry (MS) and nuclear magnetic resonance (NMR), have revealed significant metabolic alterations in skin, blood, and urine samples of psoriatic patients. Studies have identified an increase in metabolites like glutamic acid and choline in skin lesions, suggesting accelerated cell proliferation and inflammation, while a reduction in metabolites, such as glucose and lactic acid, has been observed [102].

At a systemic level, alterations in amino acids like glutamine and elevated homocysteine levels lead to intense inflammatory activity and a potential link to cardiovascular risk. These distinctive metabolic profiles not only help in understanding the disease’s pathogenesis but also hold significant potential as biomarkers. Panels of metabolites can be used for more accurate diagnosis, predicting disease severity, and monitoring treatment response [103].

Research has already shown that some treatments, like glucocorticoids and anti-TNF-α agents, induce specific changes in metabolites, offering a new perspective for personalized medicine and the identification of novel molecular biomarkers [83]. Despite encouraging mechanistic data, the clinical evidence on vitamin A and D supplementation in PSO remains fragmented. Many studies include narrowly selected patient populations or rely on short-term interventions that are insufficient to capture meaningful clinical change. Differences in dosing strategies, baseline nutritional status, and outcome measures further complicate interpretation. As a result, the current body of research provides only partial and sometimes conflicting insights, underscoring the need for more rigorous and adequately powered randomized controlled trials.

## 5. Functional and Molecular Alterations of Keratinocytes in Psoriasis: Key Signaling Pathways

In PSO, KCs undergo profound functional and molecular alterations that fuel the self-perpetuating cycle of inflammation, hyperproliferation, and defective differentiation through aberrant activation of intracellular signaling pathways that integrate inflammatory cues from immune cells and the tissue microenvironment. Among them, the NF-κB, JAK/STAT, MAPK, and PI3K/AKT/mTOR cascades, together with the IL-23/IL-17 axis, emerge as critical regulators of KC behavior [104]. Their dysregulation sustains the production of pro-inflammatory cytokines, chemokines, and antimicrobial peptides, while simultaneously promoting abnormal proliferation and impaired epidermal maturation [104]. Understanding the molecular mechanisms underlying these pathways, as well as the influence of nutrition on inflammation, provides important insights into PSO pathogenesis and highlights potential therapeutic targets. The following sections present an overview of the major signaling cascades altered in psoriatic KCs and their contribution to disease progression.

**NF-κB pathway**: As mentioned, dysregulated NF-κB signal in KCs and immune cells is a key driver in PSO pathogenesis [104]. Canonical NF-κB pathway is activated by a broad range of stimuli, including cytokines (IL-1β, IL-17A/F, IL-22, e IL-36), TNF receptor superfamily members, pattern recognition receptors (PRRs), as well as T- and B-cell receptors (TCRs and BCRs) [105]. The IKK complex (composed of IKKα, IKKβ, and regulatory subunit IKKγ) is central to the pathway [106]. Following pro-inflammatory stimulation, IKK phosphorylates IκBα, an inhibitory protein that retains NF-κB in the cytoplasm, triggering its polyubiquitination and consequent degradation; this mechanism disengages NF-κB dimers (primarily p50/RelA and p50/c-Rel), which translocate into the nucleus [106].

Dietary supplements, such as omega-3 fatty acids, polyphenols, and vitamins A, D, and E have been shown to dampen NF-κB activation by reducing IKK-mediated IκBα phosphorylation and subsequent nuclear translocation of NF-κB dimers, thereby lowering pro-inflammatory cytokine production, thus exerting anti-inflammatory response [107].

Activation of the IKK complex in the canonical pathway is tightly regulated by TRAF proteins and E3 ubiquitin ligases [106]. Notably, TRAF6 catalyzes the ubiquitination of adaptor proteins such as ACT1, thereby initiating a phosphorylation cascade that culminates in IKK activation [108]. This mechanism is critically involved in the pathogenesis of PSO. In contrast, the non-canonical NF-κB pathway relies on the NIK (NF-κB-inducing kinase)-dependent phosphorylation of p100, which leads to its processing into p52 [108]. This pathway is selectively triggered by specific members of the TNF cytokine family, such as the CD40 ligand and the B-cell activating factor (BAFF) [108].

**JAK-STAT pathway:** JAK-STAT pathway is known to play an essential role in PSO [12]. The JAK family consists of intracellular kinases, including JAK1, JAK2, JAK3, and TYK2, which associate with cytokine receptors [109]. Upon cytokine binding to their receptors, JAKs become activated and phosphorylate STAT proteins, which then dimerize and translocate into the nucleus to regulate the transcription of numerous inflammatory mediators, leading to pro-inflammatory responses [109]. JAKs mediate signaling downstream of cytokines such as IL-12, IL-23, IFN-α, IFN-β, IFN-γ, IL-6, IL-22, and others [110]. TYK2 transduces signals downstream of IL-23, IL-12, and type I IFNs [111]. JAK1, JAK2, and TYK2 are especially implicated in PSO, as they are strongly associated with the activation of the transcription factor STAT3 [111], which is hyperactivated in both KCs and immune cells, regulating key processes such as proliferation, apoptosis, and differentiation. Moreover, JAK1/JAK2-dependent signaling also contributes to STAT1 phosphorylation [12]. The increased phosphorylation of STAT1 and STAT3 observed in KCs from psoriatic lesions is critical for disease development [12]. Interestingly, the same dietary supplements that may play an effect on NF-κB may also modulate the JAK-STAT pathway [112]. Moreover, curcumin and resveratrol may inhibit JAK activation and STAT1/3 phosphorylation, thus attenuating KC hyperproliferation and Th1/Th17-mediated inflammation [112,113].

**MAPK pathway**: MAPKs are serine-threonine protein kinases also involved in the pathogenesis of PSO, regulating KC proliferation as well as immune response [114]. The MAPK family includes p38 MAPK, ERK, and JNK [115]. Each MAPK pathway is organized in a three-tier cascade: a MAPKKK activates a MAPKK through phosphorylation, and the MAPKK in turn phosphorylates and activates the downstream MAPK [115]. ERK1/2, p38, and JNK MAPK are abundantly activated in psoriatic lesions [116]. It has been found that p38 pathway, induced by the IL-17 and TNF-α, is central in PSO, regulating S100A8, hBD-2, hBD-3, S100A7 [117,118] and IL-1β production in KCs [119]. MSK1, downstream of p38/ERK1/2, regulates the expression of pro-inflammatory cytokine genes by activating transcription factors and is hyperactivated in lesions [120]. IL-6 enhances ERK1/2 activity, while KRT16 drives KCs proliferation and VEGF release via ERK [121,122]. DUSP1, a member of the phosphatase family, is an inhibitor of MAPK pathway. It is significantly downregulated in PSO patients while its overexpression limits proliferation and induces apoptosis in KCs by targeting ERK/Elk-1/Egr-1 pathway [123]. The JNK pathway in KCs can be triggered by signals such as DAMPs, CCN1, and IL-22 [123]. Once activated, it drives the production of cytokines and chemokines including IL-6, IL-8, IL-23, IFN-γ, TNF-α, and CCL20, thereby recruiting various immune cell populations into psoriatic lesions [124]. Beyond its effects on KCs proliferation and differentiation, JNK activation also promotes Th1 and Th17 cell recruitment and activation, enhancing the release of cytokines such as IL-17, IL-22, and hβD-2 [124]. Furthermore, JNK has also been identified as an important regulator of FOXP3, influencing the development and maturation of Tregs [125]. The MAPK pathway is responsive to nutritional interventions; bioactive dietary compounds, including polyphenols, flavonoids, and antioxidants can suppress p38, ERK, and JNK phosphorylation, thus reducing IL-1β, IL-6, and S100 protein expression in KCs, thereby mitigating the inflammatory and hyperproliferative processes characteristic of psoriatic lesions [126,127].

**PI3K/AKT/mTOR pathway**: Elevated expression and activation of PI3K and AKT have been observed in KCs of psoriatic lesions [128]. PI3K/Akt activation by a wide range of stimuli contributes to epidermal hyperplasia, immune dysregulation, angiogenesis, and other PSO-related processes [122]. Once activated, PI3K converts PIP2 into PIP3 at the plasma membrane, enabling Akt recruitment via its PH domain [129]. Akt is subsequently phosphorylated at Thr308 by PDK1 and at Ser473 by PDK2, leading to its full activation [129]. Activated Akt then translocates to the cytoplasm and nucleus, where its key downstream effectors, FOXO and mTOR, regulate KC proliferation and survival [129]. Elevated PI3K activity drives Akt hyperactivation, which phosphorylates FOXO. In normal skin, FOXO is nuclear, whereas in psoriatic KCs it is mainly cytoplasmic [130]. This Akt-dependent translocation reduces FOXO’s inhibitory function, thereby enhancing KCs proliferation [130]. The mTOR pathway is a key regulator of inflammation and cell proliferation in PSO. mTORC1 is activated by cytokines such as IL-1β, TNF-α, IL-17A, or IL-22, promoting KCs growth [131]. PI3K/Akt/mTOR axis also modulates innate and adaptive immunity, influencing the Th1/Th2/Th17 balance [132]. mTOR drives KCs secretion of pro-inflammatory mediators, including CXCL8, IL-6, and VEGF, while mTORC2 contributes to FOXP3 stability via CCL3 [133,134]. Furthermore, overactivated mTORC1 is linked to parakeratosis, a severe pathological lesional feature of PSO [135]. Dietary components, including polyphenols and some amino acids, can downregulate PI3K/Akt/mTOR signaling, restoring FOXO nuclear localization and limiting KC proliferation together with the modulation of mTOR-driven cytokine secretion, thus exerting significant effects on KCs and PSO [136].

**IL-17/IL-23 axis**: Intercellular communication between immune cells and KCs is central to PSO pathogenesis. KC-derived cytokines and receptors are increasingly recognized as key modulators in disease progression [137]. The IL-23/IL-17 cytokine axis is recognized as a central driver in the pathogenesis of PSO [137]. IL-23, primarily from immune cells, sustains IL-17-producing cells, but KCs also produce IL-23 [138]. Recent mouse studies show KCs-derived IL-23 alone can activate IL-17-secreting cells and drive chronic skin inflammation, with its expression regulated epigenetically via H3K9 demethylation, implicating a role in PSO [139]. The IL-17 cytokine family includes six members (IL-17A–F), with IL-17A being the key effector downstream of IL-23 in PSO [140]. The IL-17 receptor family comprises five members (IL-17RA–RE), with IL-17RA serving as the primary co-receptor for IL-17A, IL-17C, IL-17E, and IL-17F [141]. IL-17A stimulates KCs to produce antimicrobial peptides, chemokines, and pro-inflammatory cytokines, amplifying the IL-23/IL-17 axis and promoting epidermal hyperplasia via IL-19 and IL-36 [142]. Other family members, notably IL-17E and IL-17C, are also upregulated in psoriatic skin [143]; IL-17E, produced by both immune cells and KCs, drives KCs proliferation and inflammation via IL-17RB/STAT3 signaling, while IL-17C, primarily KCs-derived, forms self-amplifying inflammatory circuits and contributes to KC hyperproliferation [143], making both potential therapeutic targets in PSO and other inflammatory skin diseases. IL-22, major downstream cytokine of IL-23, mainly produced by CD4^+^ T cells and ILC3, signals through IL-22R on KCs and other epithelial cells [144]. In PSO, IL-22 drives disease by inhibiting KCs terminal differentiation and inducing antimicrobial peptides and pro-inflammatory chemokines [144]. Its activity is naturally restrained by IL-22BP, and IL-24 acts downstream of IL-22 to regulate KCs differentiation [12]. Nutrients can indirectly influence the IL-23/IL-17 axis by altering gut and systemic immunity; for instance, vitamin D and n-3 fatty acids are able to reduce IL-23 and IL-17 production and KC activation, with a positive effect on epidermal hyperplasia and inflammation, and consequently on PSO [145]. Crucially, the regulation of the inflammatory cascades discussed so far is intimately linked to the cellular metabolic status, as detailed next.

## 6. ROS and Lipid Peroxidation in Psoriasis

PSO is deeply influenced by the interplay between ROS and lipid metabolism. Excessive ROS production in immune cells and KCs leads to oxidative stress, fueling chronic inflammation [146]. Elevated ROS levels in dendritic cells promote the release of pro-inflammatory cytokines such as TNF-α and IL-8, enhancing T-cell activation and Th1 differentiation. Similarly, neutrophils and lymphocytes from psoriatic patients show increased ROS generation and lipid peroxidation, with neutrophils undergoing ROS-dependent NETosis, further amplifying KCs and immune activation [147]. The consequences of ROS excess extend to lipid peroxidation products and other mediators. Reactive aldehydes like 4-hydroxynonenal (4-HNE) and malondialdehyde (MDA), detected at high levels in psoriatic skin and plasma, can form protein adducts that disrupt cellular function [148].

Eicosanoids derived from polyunsaturated fatty acids also play a pivotal role, with omega-6–derived pro-inflammatory metabolites outweighing the protective omega-3–derived counterparts. In addition, the endocannabinoid system is altered, with complex receptor-specific effects that suggest both protective and pro-inflammatory roles. Together, ROS and lipid mediators sustain the inflammatory microenvironment of PSO and may activate adaptive antioxidant responses, such as the Nrf2 pathway [149].

Mitochondria are central regulators of redox balance, and their dysfunction is a hallmark of psoriatic lesions. An altered NADH/NAD+ ratio, reflecting a shift toward an oxidative state, has been identified as a key factor in disease pathophysiology. A novel non-invasive diagnostic tool, Flow-Mediated Skin Fluorescence (FMSF), enables dynamic assessment of NADH autofluorescence in response to ischemia–reperfusion and has demonstrated mitochondrial impairment in psoriatic skin [76].

Restoring NAD+ levels in lesions has shown promising effects, with topical NAD+ application achieving outcomes comparable to conventional treatments. This benefit is likely mediated by NAD+-dependent enzymes such as Sirtuins, particularly SIRT1 [150].

Targeting mitochondrial metabolism and redox imbalance thus emerges as a novel therapeutic strategy, with FMSF offering a translational bridge for both diagnosis and personalized treatment monitoring. The resulting metabolic and redox imbalance requires fine molecular control. In this context, Sirtuins emerge as critical sensors

## 7. Sirtuins and Redox-Dependent Pathways

Sirtuins, particularly SIRT1, are critical regulators of oxidative stress and inflammation in PSO. They maintain cellular balance by integrating redox control, metabolism, and immune regulation [151]. Mechanistically, SIRT1 suppresses KC hyperproliferation and migration by deacetylating STAT3, and it counteracts inflammation by deacetylating the p65 subunit of NF-κB, thereby reducing pro-inflammatory cytokine release (TNF-α, IL-6) [152].

Importantly, NF-κB can downregulate SIRT1 expression, creating a vicious cycle of chronic inflammation [149,150]. SIRT1 activity is closely linked to the AMPK pathway, a master regulator of cellular energy metabolism. In psoriatic skin, AMPK activity is often diminished; however, its activation enhances NAD+ availability and, consequently, SIRT1 activity [150,153]. This reciprocal regulation suggests that dual targeting of AMPK/NAD+/SIRT1 axis could restore KC homeostasis and dampen inflammation representing a promising strategy [151]. Building on this understanding, therapeutic strategies focus on manipulating SIRT1 activity. For example, resveratrol (RSV), a potent natural activator of SIRT1, exerts anti-inflammatory effects by inhibiting the NF-κB pathway, reducing inflammatory cytokines, and promoting KCs apoptosis [150]. Similarly, catalpol, an iridoid glucoside, increases SIRT1 expression and blocks inflammatory pathways such as NF-κB and MAPK [154].

Targeting mitochondrial metabolism and redox imbalance thus emerges as a novel therapeutic strategy [65,155]. By controlling these intricate signaling networks, modulation of sirtuins and their downstream pathways represents a promising frontier for personalized treatments for PSO [149,152].

## 8. Conclusions

PSO represents a complex interplay between immune dysregulation, KCs dysfunction, and metabolic imbalances. KCs emerge as central orchestrators of inflammation, responding to cytokines, nutrients, metabolites, and signals derived from the gut–skin axis, where intestinal microbiota composition and their metabolites, such as SCFAs, modulate both cutaneous and systemic inflammation. Key intracellular signaling pathways, including NF-κB, JAK/STAT, MAPK, and PI3K/AKT/mTOR, together with the IL-23/IL-17 axis, drive epidermal hyperproliferation and pro-inflammatory mediator production. Metabolic and redox status, including sirtuin activity and the NAD+/AMPK balance, further shapes cellular homeostasis and offers potential therapeutic targets [155].

Our review specifically integrated these diverse factors, proposing that the KCs metabolic state, particularly via the Sirtuin/NAD+ axis, serves as a crucial convergence point where systemic nutritional and microbial signals meet intrinsic inflammatory pathways. This synthesis provides a mechanistic rationale for shifting therapeutic focus toward metabolic checkpoints.

Nutritional and microbiota-based modulation, combined with targeted intervention on key signaling pathways, represents a promising approach to personalize PSO management, reduce chronic inflammation, and restore epidermal homeostasis [156].

## 9. Future Directions

Emerging evidence suggests that targeting KCs could provide new opportunities for the treatment of various skin diseases. Future research should focus on translating these insights into clinical practice, exploring personalized approaches that combine microbiota-based interventions with nutraceutical strategies to support skin health. In addition, further studies are warranted to translate these mechanistic insights into safe and effective clinical interventions, integrating molecular, metabolic, and microbial knowledge for precision medicine in PSO. Such efforts may enable innovative, non-invasive therapies that support existing treatments, as dietary supplements and/or adjuvant therapy, and improve patient adherence and outcomes.

## Figures and Tables

**Table 1:** Summary of key SCFA-mediated mechanisms along the gut–skin axis relevant to PSO.

Mechanism	Cell Types Involved	Functional Effects	Relevance in PSO
GPCR activation	IECs, DCs, macrophages, T cells	↑ IL-10, ↓ IL-17; regulation of neutrophil chemotaxis; promotion of Treg development	Reduced GPR43/109A expression in psoriatic skin; restored by butyrate
HDAC inhibition	IECs, DCs, KCs, T cells	↑ Histone acetylation; repression of TNF-α, IL-6; ↑ barrier proteins (filaggrin, TGM-1)	Corrects aberrant HDAC activity seen in psoriatic lesions; improves barrier integrity
Promotion of retinoic acid synthesis	Gut DCs	↑ Foxp3 expression; stabilization of Treg phenotype	Restores defective Treg responses in psoriatic inflammation
Modulation of Treg/Th17 axis	Tregs, Th17 cells, DCs	↑ Tregs, ↓ Th17 cells; normalization of IL-17 pathways	Central for correcting the Th17-skewed immune profile in PSO
Regulation of DC function	DCs	Enhanced antigen uptake; altered cytokine production; ↑ RA synthesis	Reduces pathogenic DC activity driving Th17 responses
Effects on KCs	KCs	↑ Filaggrin/TGM-1; ↓ oxidative stress; improved mitochondrial function	Counteracts hyperproliferation and barrier dysfunction in plaques
Breg induction	Regulatory B cells	↑ IL-10-producing Bregs; ↑ IgA/IgG secretion	Dampens IL-23/Th17 axis, supports immune tolerance
Gut barrier protection	IECs	↓ permeability; ↓ LPS translocation	Reduces TLR4 activation and downstream psoriatic inflammation

IECs: intestinal epithelial cells; DCs: dendritic cells; Tregs: regulatory T cells; Th17: T helper 17 cells; Bregs: regulatory B cells; GPCR: G-protein-coupled receptor; GPR43/109A: G-protein-coupled receptor 43/hydroxycarboxylic acid receptor 2; HDAC: histone deacetylase; RA: retinoic acid; TGM-1: transglutaminase-1; IL: interleukin; TNF-α: tumor necrosis factor alpha; LPS: lipopolysaccharide; ↑ increase; ↓ decrease.

**Table 2:** Microbiome–PSO associations: protective vs. risk taxa with hypothesized mechanisms.

Taxon (Level)	Effect	Level of Evidence	Expected/Principal Mechanism	Potential Therapeutic Lever	References
*Prevotella* (genus)	↑ risk	Cohorts	SCFA-profile shift; Th17 activation	High-fiber targeted diet; pre/probiotics	Sonomoto et al., 2023 [60]
*Lachnospira* (genus)	↓ protective	Cohorts	SCFA producer → ↑ Treg, barrier support	Increase fermentable fibers; prebiotics	Sonomoto et al., 2023 [60]
*Akkermansia muciniphila* (species)	↓ protective	Cohorts	Mucus-layer/barrier integrity	Targeted pre/probiotics; lifestyle	Sonomoto et al., 2023 [60]
*Lentimicrobiaceae* (family)	Protective	MR	Immunomodulation (hypothesized)	––	Qian et al., 2024 [58]
*Alistipes* (genus)	Protective	Cohorts + plausibility	SCFA production → ↓ cytokines, ↑ Treg	––	Scher et al., 2015 [66]; Hidalgo-Cantabrana et al., 2019 [67]; Parker et al., 2020 [68]
*Lactobacillus salivarius* (species)	Protective	Preclinical/early clinical	Bacteriocins; barrier reinforcement	Strain-selective probiotics	Messaoudi et al., 2013 [69]
*Clostridia* CAG-485/CAG-83 (species)	Protective	MR/signals	Th1/Th2/Th17 rebalance; ↑ Treg	Probiotics	Chen et al., 2020; [53] Qian et al., 2024 [58]
*Helicobacter* (genus)	Protective signal in Qian et al., 2024 [58]; H. pylori controversial	Cohorts/meta-analysis	Species-dependent	––	Yu et al., 2019 [63]; Fabrizi et al., 2001 [64]; Azizzadeh et al., 2014 [65]
*Omnitrophota*/*Omnitrophica* (phylum)	↑ risk	MR/observational	Unknown	––	Seymour et al., 2023 [70]; Qian et al., 2024 [58]
*Flavobacteriales* (order)	↑ risk	MR/observational	Immuno-activation (hypothesized)	––	Qian et al., 2024 [58]
*Elusimicrobiaceae* (family)	↑ risk	MR/observational	Barrier/metabolite dysfunction (hypothesized)	––	Qian et al., 2024 [58]
*Fusobacteriaceae* (family)	↑ risk	Cohorts/models	Pro-inflammatory	––	Keku et al., 2013 [71]; Rau et al., 2018 [72]
CAG-698 (family)	↑ risk	MR	Not defined	––	Qian et al., 2024 [58]
CAG-977 (family)	↑ risk; bidirectional signal	MR	Not defined	––	Qian et al., 2024 [58]
*Bacillus* AY (genus)	↑ risk	MR	Not defined	––	Qian et al., 2024 [58]
*Brevibacillus* B (genus)	↑ risk	MR	Not defined	––	Qian et al., 2024 [58]
*Demequina* (genus)	↑ risk	MR	Not defined	––	Qian et al., 2024 [58]
UBA6398 (genus)	↑ risk	MR/observational	Not defined	––	Qian et al., 2024 [58]
*Blautia* sp001304935 (species; *Lachnospiraceae*)	↑ risk	MR	Pro-inflammatory metabolites (hypothesized)	––	Vacca et al., 2020 [62]; Chen et al., 2018 [73]; Hidalgo-Cantabrana et al., 2019 [67]; Sun et al., 2021 [74]
*Desulfovibrio piger* (species)	↑ risk	Mechanistic	H_2_S production → mucosal damage	––	Rey et al., 2013 [54]

↑ increase; ↓ decrease; → lead/induce.

## Data Availability

No new data were created or analyzed in this study. Data sharing is not applicable to this article.

## Abbreviations

4-HNE	4-Hydroxynonenal
ACT1	Aktivating Characteristic of TRAF-binding protein 1
AMPK	Adenosine Monophosphate-Protein Kinase
BAFF	B-cellActivating Factor
BCRs	B-Cell Receptors
Bregs	B regulatory cells
C1, C2, C3, C4, C5, C6	Carbon atoms
CAG	Cluster of Associated Genes
CCL20	C-C Ligand 20
CCR6	C-C Receptor type 6
CD4^+^, CD8^+^, CD25^+^	Cluster of Differentiation
CRP	C-Reactive Protein
CXCL-	Chemokine Ligand
DAMPs	Danger-Associated Molecular Patterns
DCs	Dendritic Cells
DHA	Docosahexaenoic Acid
DUSP1	Dual-Specificity Phosphatase 1
EPA	Eicosapentaenoic Acid
ERK	Extracellular-signal Regulated Kinase
FMSF	Flow-Mediated Skin Fluorescence
FOXO	Forkhead O box O
GI	Glycemic Index
GL	Glycemic Load
GPCR	G-Protein-Coupled Receptor
GPR109A (HCAR2)	G-Protein-Receptor 109A (Hydroxycarboxylic Acid Receptor 2)
GPR43	G-Protein-Receptor 43
GWAS	Genome-Wide Association Studies
H.	Helicobacter
HbA1c	Hemoglobin A1c
hBD2	human Beta-Defensin 2
hBD-2, hBD-3	human Beta-Defensin 2, 3
HCA2	Hydroxycarboxylic Acid Receptor 2
HDAC	Histone Deacetylase
hsCRP	high-sensitivity C-Reactive Protein
IgA, IgG	Immunoglobulin A, G
IKK	IκB Kinase
IL-	Interleukin
IL-17RA–RE	Interleukin 17 Receptor A–E
IL-22BP	Interleukin 22 Binding Protein
ILC3	Innate Lymphoid Cell Type 3
IMQ	Imiquimod
IFN-γ	Interferon gamma
JAK/STAT	Janus Kinase/Signal Transducers and Activators of Transcription
JNK	Jun N-terminal Kinase
KC	Keratinocyte
KRT16	Keratin 16
LPS	Lipopolysaccharide
MAPK	Mitogen-Activated Protein Kinase
mDCs	myeloid Dendritic Cells
MDA	Malondialdehyde
MR	Mendelian Randomization
MS	Mass Spectrometry
MSK1	Mitogen-and Stress-activated protein Kinase 1
mTOR	mammalian Target of Rapamycin
mTORC1/mTORC2	mTOR Complex 1/2
NAD+/NADH	Nicotinamide Adenine Dinucleotide
n-3 FAs (Omega-3s)	N-3 Fatty Acids
n-6 FAs (Omega-6s)	N-6 Fatty Acids
NF-κB	Nuclear Factor kappa-light-chain-enhancer of activated B cells
NIK	NF-κB-inducing kinase
NLRP3	NLR pyrin 3
NMR	Nuclear Magnetic Resonance
NO	Nitric Oxide
PASI	Psoriasis Area and Severity Index
pDCs	plasmacytoid Dendritic Cells
PDK1/PDK2	Phosphoinositide-dependent Kinase 1/2
PH domain	Pleckstrin Homology domain
PI3K/AKT/mTOR	Phosphoinositide 3-Kinase/AKT/mTOR
PIP2/PIP3	Phosphatidylinositol (4,5)-bisphosphate/(3,4,5)-trisphosphate
PPAR-γ	Peroxisome Proliferator-Activated Receptor gamma
PRRs	Pattern Recognition Receptors
PSO	Psoriasis
p38-MAPK	p38 Mitogen-Activated Protein Kinase
PUFAs	Polyunsaturated Fatty Acids
ROS	Reactive Oxygen Species
RSV	Resveratrol
SAA	Serum Amyloid A
SCFAs	Short Chain Fatty Acids
SFAs	Saturated Fatty Acids
STAT	Signal Transducer and Activator of Transcription
TCA cycle	Tricarboxylic Acid cycle
TCRs	T-Cell Receptors
TCV	Total Caloric Value
TGF-β1	Transforming Growth Factor beta 1
Th1	T helper type 1 cell
Th2	T helper type 2 cell
Th17	T helper type 17 cell
TLR4	Toll-like Receptor 4
TMA	Trimethylamine
TMAO	Trimethylamine N-oxide
TNF-α	Tumor Necrosis Factor alpha
TOR/PI3K/MAPK	Target of Rapamycin/PI3K/MAPK
TRAF	TNF Receptor-Associated Factor
Tregs	T regulatory cells
TYK2	Tyrosine Kinase 2
UVB	Ultraviolet B
VEGF	Vascular Endothelial Growth Factor
VLCKDs	Very Low-Calorie Ketogenic Diets
γδ T cells	Gamma-delta T cells

## References

[1] Rendon A., Schäkel K. (2019). Psoriasis Pathogenesis and Treatment. Int. J. Mol. Sci..

[2] Armstrong A.W., Read C. (2020). Pathophysiology, Clinical Presentation, and Treatment of Psoriasis: A Review. JAMA.

[3] Peters B.P., Weissman F.G., Gill M.A. (2000). Pathophysiology and treatment of psoriasis. Am. J. Health-Syst. Pharm. AJHP Off. J. Am. Soc. Health-Syst. Pharm..

[4] Todke P., Shah V.H. (2018). Psoriasis: Implication to disease and therapeutic strategies, with an emphasis on drug delivery approaches. Int. J. Dermatol..

[5] Valenzuela F., Flores R. (2022). Clinical Overview of Psoriasis and Psoriatic Arthritis. Textbook of Dermatologic Ultrasound.

[6] Greb J.E., Goldminz A.M., Elder J.T., Lebwohl M.G., Gladman D.D., Wu J.J., Mehta N.N., Finlay A.Y., Gottlieb A.B. (2016). Psoriasis. Nat. Rev. Dis. Primers.

[7] Takeshita J., Grewal S., Langan S.M., Mehta N.N., Ogdie A., Van Voorhees A.S., Gelfand J.M. (2017). Psoriasis and comorbid diseases: Epidemiology. J. Am. Acad. Dermatol..

[8] Barrea L., Balato N., Di Somma C., Macchia P.E., Napolitano M., Savanelli M.C., Esposito K., Colao A., Savastano S. (2015). Nutrition and psoriasis: Is there any association between the severity of the disease and adherence to the Mediterranean diet?. J. Transl. Med..

[9] Gooderham M.J., Papp K.A., Lynde C.W. (2018). Shifting the focus—The primary role of IL-23 in psoriasis and other inflammatory disorders. J. Eur. Acad. Dermatol. Venereol. JEADV.

[10] Hawkes J.E., Yan B.Y., Chan T.C., Krueger J.G. (2018). Discovery of the IL-23/IL-17 Signaling Pathway and the Treatment of Psoriasis. J. Immunol..

[11] Lee Y.G., Jung Y., Choi H.-K., Lee J.-I., Lim T.-G., Lee J. (2024). Natural Product-Derived Compounds Targeting Keratinocytes and Molecular Pathways in Psoriasis Therapeutics. Int. J. Mol. Sci..

[12] Zhou X., Chen Y., Cui L., Shi Y., Guo C. (2022). Advances in the pathogenesis of psoriasis: From keratinocyte perspective. Cell Death Dis..

[13] Sieminska I., Pieniawska M., Grzywa T.M. (2024). The Immunology of Psoriasis-Current Concepts in Pathogenesis. Clin. Rev. Allergy Immunol..

[14] Perera G.K., Di Meglio P., Nestle F.O. (2012). Psoriasis. Annu. Rev. Pathol..

[15] Mabuchi T., Takekoshi T., Hwang S.T. (2011). Epidermal CCR6+ γδ T cells are major producers of IL-22 and IL-17 in a murine model of psoriasiform dermatitis. J. Immunol..

[16] Xu M., Lu H., Lee Y.H., Wu Y., Liu K., Shi Y., An H., Zhang J., Wang X., Lai Y. (2018). An Interleukin-25-Mediated Autoregulatory Circuit in Keratinocytes Plays a Pivotal Role in Psoriatic Skin Inflammation. Immunity.

[17] Nestle F.O., Kaplan D.H., Barker J. (2009). Psoriasis. N. Engl. J. Med..

[18] Lowes M.A., Russell C.B., Martin D.A., Towne J.E., Krueger J.G. (2013). The IL-23/T17 pathogenic axis in psoriasis is amplified by keratinocyte responses. Trends Immunol..

[19] Hawkes J.E., Chan T.C., Krueger J.G. (2017). Psoriasis pathogenesis and the development of novel targeted immune therapies. J. Allergy Clin. Immunol..

[20] Martínez J.E., Vargas A., Pérez-Sánchez T., Encío I.J., Cabello-Olmo M., Barajas M. (2021). Human Microbiota Network: Unveiling Potential Crosstalk between the Different Microbiota Ecosystems and Their Role in Health and Disease. Nutrients.

[21] Opazo M.C., Ortega-Rocha E.M., Coronado-Arrázola I., Bonifaz L.C., Boudin H., Neunlist M., Bueno S.M., Kalergis A.M., Riedel C.A. (2018). Intestinal Microbiota Influences Non-intestinal Related Autoimmune Diseases. Front. Microbiol..

[22] Honda K., Littman D.R. (2016). The microbiota in adaptive immune homeostasis and disease. Nature.

[23] Muscolino P., Granata B., Omero F., De Pasquale C., Campana S., Calabrò A., D’Anna F., Drommi F., Pezzino G., Cavaliere R. (2023). Potential predictive role of gut microbiota to immunotherapy in HCC patients: A brief review. Front. Oncol..

[24] Chen M., Wang R., Wang T. (2024). Gut microbiota and skin pathologies: Mechanism of the gut-skin axis in atopic dermatitis and psoriasis. Int. Immunopharmacol..

[25] Zhang Q., Zhao L., Li Y., Wang S., Lu G., Wang H. (2024). Advances in the mechanism of action of short-chain fatty acids in psoriasis. Int. Immunopharmacol..

[26] Andrani M., Borghetti P., Ravanetti F., Cavalli V., Ferrari L., De Angelis E., Martelli P., Saleri R. (2023). Acetate and propionate effects in response to LPS in a porcine intestinal co-culture model. Porc. Health Manag..

[27] Fallahi N., Rafiee M., Hosseini S.S., Sereshki N., Anani Sarab G., Erfanian N. (2025). Short-chain fatty acids and their role in modulating autoimmune responses in psoriasis: Insights from recent microbiota research. Lett. Appl. Microbiol..

[28] Haghikia A., Zimmermann F., Schumann P., Jasina A., Roessler J., Schmidt D., Heinze P., Kaisler J., Nageswaran V., Aigner A. (2022). Propionate attenuates atherosclerosis by immune-dependent regulation of intestinal cholesterol metabolism. Eur. Heart J..

[29] Zheng L., Kelly C.J., Battista K.D., Schaefer R., Lanis J.M., Alexeev E.E., Wang R.X., Onyiah J.C., Kominsky D.J., Colgan S.P. (2017). Microbial-Derived Butyrate Promotes Epithelial Barrier Function through IL-10 Receptor-Dependent Repression of Claudin-2. J. Immunol..

[30] Seljeset S., Siehler S. (2012). Receptor-specific regulation of ERK1/2 activation by members of the “free fatty acid receptor” family. J. Recept. Signal Transduct. Res..

[31] Nadeem A., Ahmad S.F., Al-Harbi N.O., El-Sherbeeny A.M., Al-Harbi M.M., Almukhlafi T.S. (2017). GPR43 activation enhances psoriasis-like inflammation through epidermal upregulation of IL-6 and dual oxidase 2 signaling in a murine model. Cell Signal.

[32] Shi W., Tan H., Liao C., An Z. (2025). Cross-regulation between adipose tissue innervation and metaflammation: A potential therapeutic target for obesity. Am. J. Transl. Res..

[33] Krejner A., Bruhs A., Mrowietz U., Wehkamp U., Schwarz T., Schwarz A. (2018). Decreased expression of G-protein-coupled receptors GPR43 and GPR109a in psoriatic skin can be restored by topical application of sodium butyrate. Arch. Dermatol. Res..

[34] Schwarz A., Philippsen R., Piticchio S.G., Hartmann J.N., Häsler R., Rose-John S., Schwarz T. (2023). Crosstalk between microbiome, regulatory T cells and HCA2 orchestrates the inflammatory response in a murine psoriasis model. Front. Immunol..

[35] He J., Zhang P., Shen L., Niu L., Tan Y., Chen L., Zhao Y., Bai L., Hao X., Li X. (2020). Short-Chain Fatty Acids and Their Association with Signalling Pathways in Inflammation, Glucose and Lipid Metabolism. Int. J. Mol. Sci..

[36] Tovar-Castillo L.E., Cancino-Díaz J.C., García-Vázquez F., Cancino-Gómez F.G., León-Dorantes G., Blancas-González F., Jiménez-Zamudio L., García-Latorre E., Cancino-Díaz M.E. (2007). Under-expression of VHL and over-expression of HDAC-1, HIF-1alpha, LL-37, and IAP-2 in affected skin biopsies of patients with psoriasis. Int. J. Dermatol..

[37] Hwang Y.J., Na J.I., Byun S.Y., Kwon S.H., Yang S.H., Lee H.S., Choi H.R., Cho S., Youn S.W., Park K.C. (2020). Histone Deacetylase 1 and Sirtuin 1 Expression in Psoriatic Skin: A Comparison between Guttate and Plaque Psoriasis. Life.

[38] Luu M., Riester Z., Baldrich A., Reichardt N., Yuille S., Busetti A., Klein M., Wempe A., Leister H., Raifer H. (2021). Microbial short-chain fatty acids modulate CD8(+) T cell responses and improve adoptive immunotherapy for cancer. Nat. Commun..

[39] Leon Carrion S., Sutter C.H., Sutter T.R. (2014). Combined treatment with sodium butyrate and PD153035 enhances keratinocyte differentiation. Exp. Dermatol..

[40] Inamoto T., Furuta K., Han C., Uneme M., Kano T., Ishikawa K., Kaito C. (2023). Short-chain fatty acids stimulate dendrite elongation in dendritic cells by inhibiting histone deacetylase. FEBS J..

[41] Stec A., Sikora M., Maciejewska M., Paralusz-Stec K., Michalska M., Sikorska E., Rudnicka L. (2023). Bacterial Metabolites: A Link between Gut Microbiota and Dermatological Diseases. Int. J. Mol. Sci..

[42] Kaisar M.M.M., Pelgrom L.R., van der Ham A.J., Yazdanbakhsh M., Everts B. (2017). Butyrate Conditions Human Dendritic Cells to Prime Type 1 Regulatory T Cells via both Histone Deacetylase Inhibition and G Protein-Coupled Receptor 109A Signaling. Front. Immunol..

[43] Kaye D.M., Shihata W.A., Jama H.A., Tsyganov K., Ziemann M., Kiriazis H., Horlock D., Vijay A., Giam B., Vinh A. (2020). Deficiency of Prebiotic Fiber and Insufficient Signaling Through Gut Metabolite-Sensing Receptors Leads to Cardiovascular Disease. Circulation.

[44] Luu M., Pautz S., Kohl V., Singh R., Romero R., Lucas S., Hofmann J., Raifer H., Vachharajani N., Carrascosa L.C. (2019). The short-chain fatty acid pentanoate suppresses autoimmunity by modulating the metabolic-epigenetic crosstalk in lymphocytes. Nat. Commun..

[45] Gao Y., Xu T., Wang Y., Hu Y., Yin S., Qin Z., Yu H. (2025). Pathophysiology and Treatment of Psoriasis: From Clinical Practice to Basic Research. Pharmaceutics.

[46] Wang X., Yao Y., Li Y., Guo S., Li Y., Zhang G. (2023). Experimental study on the effect of luteolin on the proliferation, apoptosis and expression of inflammation-related mediators in lipopolysaccharide-induced keratinocytes. Int. J. Immunopathol. Pharmacol..

[47] Jimenez-Sanchez M., Celiberto L.S., Yang H., Sham H.P., Vallance B.A. (2025). The gut-skin axis: A bi-directional, microbiota-driven relationship with therapeutic potential. Gut Microbes.

[48] Pachauri A., Sharma S. (2025). Unravelling the gut-skin axis: The role of gut microbiota in pathogenesis and management of psoriasis. Inflammopharmacology.

[49] Du H.X., Yue S.Y., Niu D., Liu C., Zhang L.G., Chen J., Chen Y., Guan Y., Hua X.L., Li C. (2022). Gut Microflora Modulates Th17/Treg Cell Differentiation in Experimental Autoimmune Prostatitis via the Short-Chain Fatty Acid Propionate. Front. Immunol..

[50] Johnson-Huang L.M., Suárez-Fariñas M., Sullivan-Whalen M., Gilleaudeau P., Krueger J.G., Lowes M.A. (2010). Effective narrow-band UVB radiation therapy suppresses the IL-23/IL-17 axis in normalized psoriasis plaques. J. Investig. Dermatol..

[51] Mahmud M.R., Akter S., Tamanna S.K., Mazumder L., Esti I.Z., Banerjee S., Akter S., Hasan M.R., Acharjee M., Hossain M.S. (2022). Impact of gut microbiome on skin health: Gut-skin axis observed through the lenses of therapeutics and skin diseases. Gut Microbes.

[52] Widhiati S., Purnomosari D., Wibawa T., Soebono H. (2022). The role of gut microbiome in inflammatory skin disorders: A systematic review. Dermatol. Rep..

[53] Chen L., Li J., Zhu W., Kuang Y., Liu T., Zhang W., Chen X., Peng C. (2020). Skin and Gut Microbiome in Psoriasis: Gaining Insight Into the Pathophysiology of It and Finding Novel Therapeutic Strategies. Front. Microbiol..

[54] Rey F.E., Gonzalez M.D., Cheng J., Wu M., Ahern P.P., Gordon J.I. (2013). Metabolic niche of a prominent sulfate-reducing human gut bacterium. Proc. Natl. Acad. Sci. USA.

[55] Buhaș M.C., Gavrilaș L.I., Candrea R., Cătinean A., Mocan A., Miere D., Tătaru A. (2022). Gut Microbiota in Psoriasis. Nutrients.

[56] Vinolo M.A., Rodrigues H.G., Hatanaka E., Sato F.T., Sampaio S.C., Curi R. (2011). Suppressive effect of short-chain fatty acids on production of proinflammatory mediators by neutrophils. J. Nutr. Biochem..

[57] Shapiro J., Cohen N.A., Shalev V., Uzan A., Koren O., Maharshak N. (2019). Psoriatic patients have a distinct structural and functional fecal microbiota compared with controls. J. Dermatol..

[58] Qian M., Shi J., Zhang Z., Bi D., Tan C. (2024). Genetic insights into the gut microbiota and risk of psoriasis: A bidirectional mendelian randomization study. Front. Microbiol..

[59] Polak K., Bergler-Czop B., Szczepanek M., Wojciechowska K., Frątczak A., Kiss N. (2021). Psoriasis and Gut Microbiome-Current State of Art. Int. J. Mol. Sci..

[60] Sonomoto K., Song R., Eriksson D., Hahn A.M., Meng X., Lyu P., Cao S., Liu N., Taudte R.V., Wirtz S. (2023). High-fat-diet-associated intestinal microbiota exacerbates psoriasis-like inflammation by enhancing systemic γδ T cell IL-17 production. Cell Rep..

[61] Xiao S., Zhang G., Jiang C., Liu X., Wang X., Li Y., Cheng M., Lv H., Xian F., Guo X. (2021). Deciphering Gut Microbiota Dysbiosis and Corresponding Genetic and Metabolic Dysregulation in Psoriasis Patients Using Metagenomics Sequencing. Front. Cell. Infect. Microbiol..

[62] Vacca M., Celano G., Calabrese F.M., Portincasa P., Gobbetti M., De Angelis M. (2020). The Controversial Role of Human Gut Lachnospiraceae. Microorganisms.

[63] Yu M., Zhang R., Ni P., Chen S., Duan G. (2019). Helicobacter pylori Infection and Psoriasis: A Systematic Review and Meta-Analysis. Medicina.

[64] Fabrizi G., Carbone A., Lippi M.E., Anti M., Gasbarrini G. (2001). Lack of evidence of relationship between Helicobacter pylori infection and psoriasis in childhood. Arch. Dermatol..

[65] Azizzadeh M., Nejad Z.V., Ghorbani R., Pahlevan D. (2014). Relationship between Helicobacter pylori infection and psoriasis. Ann. Saudi Med..

[66] Scher J.U., Ubeda C., Artacho A., M A., Isaac S., Reddy S.M., Marmon S., Neimann A., Brusca S., Patel T. (2015). Decreased Bacterial Diversity Characterizes the Altered Gut Microbiota in Patients With Psoriatic Arthritis, Resembling Dysbiosis in Inflammatory Bowel Disease. Arthritis Rheumatol..

[67] Hidalgo-Cantabrana C., Gómez J., Delgado S., Requena-López S., Queiro-Silva R., Margolles A., Coto E., Sánchez B., Coto-Segura P. (2019). Gut microbiota dysbiosis in a cohort of patients with psoriasis. Br. J. Dermatol..

[68] Parker B.J., Wearsch P.A., Veloo A.C.M., Rodriguez-Palacios A. (2020). The Genus Alistipes: Gut Bacteria With Emerging Implications to Inflammation, Cancer, and Mental Health. Front. Immunol..

[69] Messaoudi S., Manai M., Kergourlay G., Prévost H., Connil N., Chobert J.-M., Dousset X. (2013). Lactobacillus salivarius: Bacteriocin and probiotic activity. Food Microbiol..

[70] Seymour C.O., Palmer M., Becraft E.D., Stepanauskas R., Friel A.D., Schulz F., Woyke T., Eloe-Fadrosh E., Lai D., Jiao J.-Y. (2023). Hyperactive nanobacteria with host-dependent traits pervade Omnitrophota. Nat. Microbiol..

[71] Keku T.O., McCoy A.N., Azcarate-Peril A.M. (2013). *Fusobacterium* spp. and colorectal cancer: Cause or consequence?. Trends Microbiol..

[72] Rau M., Rehman A., Dittrich M., Groen A.K., Hermanns H.M., Seyfried F., Beyersdorf N., Dandekar T., Rosenstiel P., Geier A. (2018). Fecal SCFAs and SCFA-producing bacteria in gut microbiome of human NAFLD as a putative link to systemic T-cell activation and advanced disease. United Eur. Gastroenterol. J..

[73] Chen Y., Ho H.J., Tseng C., Lai Z., Shieh J., Wu C. (2018). Intestinal microbiota profiling and predicted metabolic dysregulation in psoriasis patients. Exp. Dermatol..

[74] Sun C., Chen L., Yang H., Sun H., Xie Z., Zhao B., Jiang X., Qin B., Shen Z. (2021). Involvement of Gut Microbiota in the Development of Psoriasis Vulgaris. Front. Nutr..

[75] Herbert D., Franz S., Popkova Y., Anderegg U., Schiller J., Schwede K., Lorz A., Simon J.C., Saalbach A. (2018). High-Fat Diet Exacerbates Early Psoriatic Skin Inflammation Independent of Obesity: Saturated Fatty Acids as Key Players. J. Investig. Dermatol..

[76] Gebicki J., Filipiak T., Marcinek A., Wozniacka A. (2023). Assessment of NADH/NAD^+^ Redox Imbalance in Psoriatic Lesions Using the FMSF Technique: Therapeutic Aspects. Sensors.

[77] Honda T., Kabashima K. (2019). Current understanding of the role of dietary lipids in the pathophysiology of psoriasis. J. Dermatol. Sci..

[78] Kanda N., Hoashi T., Saeki H. (2020). Nutrition and Psoriasis. Int. J. Mol. Sci..

[79] Garbicz J., Całyniuk B., Górski M., Buczkowska M., Piecuch M., Kulik A., Rozentryt P. (2021). Nutritional Therapy in Persons Suffering from Psoriasis. Nutrients.

[80] Korovesi A., Dalamaga M., Kotopouli M., Papadavid E. (2019). Adherence to the Mediterranean diet is independently associated with psoriasis risk, severity, and quality of life: A cross-sectional observational study. Int. J. Dermatol..

[81] Aryanian Z., Asghari M., Zanousi P.P., Ghadimi R., Kebria A.S., Hatami P. (2024). Adherence to the Mediterranean diet in patients with psoriasis and its relationship with the severity of the disease: A case-control study. Health Sci. Rep..

[82] Umar M., Sastry K.S., Al Ali F., Al-Khulaifi M., Wang E., Chouchane A.I. (2018). Vitamin D and the Pathophysiology of Inflammatory Skin Diseases. Skin Pharmacol. Physiol..

[83] Lowes M.A., Suárez-Fariñas M., Krueger J.G. (2014). Immunology of psoriasis. Annu. Rev. Immunol..

[84] Balbás G.M., Regaña M.S., Millet P.U. (2011). Study on the use of omega-3 fatty acids as a therapeutic supplement in treatment of psoriasis. Clin. Cosmet. Investig. Dermatol..

[85] Bulló M., Casas R., Portillo M.P., Basora J., Estruch R., García-Arellano A., Lasa A., Juanola-Falgarona M., Arós F., Salas-Salvadó J. (2013). Dietary glycemic index/load and peripheral adipokines and inflammatory markers in elderly subjects at high cardiovascular risk. Nutr. Metab. Cardiovasc. Dis. NMCD.

[86] Katsimbri P., Korakas E., Kountouri A., Ikonomidis I., Tsougos E., Vlachos D., Papadavid E., Raptis A., Lambadiari V. (2021). The Effect of Antioxidant and Anti-Inflammatory Capacity of Diet on Psoriasis and Psoriatic Arthritis Phenotype: Nutrition as Therapeutic Tool?. Antioxidants.

[87] Liu S., Manson J.E., Buring J.E., Stampfer M.J., Willett W.C., Ridker P.M. (2002). Relation between a diet with a high glycemic load and plasma concentrations of high-sensitivity C-reactive protein in middle-aged women. Am. J. Clin. Nutr..

[88] Kasim-Karakas S.E., Tsodikov A., Singh U., Jialal I. (2006). Responses of inflammatory markers to a low-fat, high-carbohydrate diet: Effects of energy intake. Am. J. Clin. Nutr..

[89] Nicklas B.J., Ambrosius W., Messier S.P., Miller G.D., Penninx B.W., Loeser R.F., Palla S., Bleecker E., Pahor M. (2004). Diet-induced weight loss, exercise, and chronic inflammation in older, obese adults: A randomized controlled clinical trial. Am. J. Clin. Nutr..

[90] O’Brien K.D., Brehm B.J., Seeley R.J., Bean J., Wener M.H., Daniels S., D’Alessio D.A. (2005). Diet-induced weight loss is associated with decreases in plasma serum amyloid a and C-reactive protein independent of dietary macronutrient composition in obese subjects. J. Clin. Endocrinol. Metab..

[91] Edrisi F., Salehi M., Ahmadi A., Fararoei M., Rusta F., Mahmoodianfard S. (2018). Effects of supplementation with rice husk powder and rice bran on inflammatory factors in overweight and obese adults following an energy-restricted diet: A randomized controlled trial. Eur. J. Nutr..

[92] Le Poul E., Loison C., Struyf S., Springael J.Y., Lannoy V., Decobecq M.E., Brezillon S., Dupriez V., Vassart G., Van Damme J. (2003). Functional characterization of human receptors for short chain fatty acids and their role in polymorphonuclear cell activation. J. Biol. Chem..

[93] Christophers E. (2001). Psoriasis--epidemiology and clinical spectrum. Clin. Exp. Dermatol..

[94] Deng Y., Chang C., Lu Q. (2016). The Inflammatory Response in Psoriasis: A Comprehensive Review. Clin. Rev. Allergy Immunol..

[95] Muscogiuri G., Barrea L., Laudisio D., Pugliese G., Salzano C., Savastano S., Colao A. (2019). The management of very low-calorie ketogenic diet in obesity outpatient clinic: A practical guide. J. Transl. Med..

[96] Phan C., Touvier M., Kesse-Guyot E., Adjibade M., Hercberg S., Wolkenstein P., Chosidow O., Ezzedine K., Sbidian E. (2018). Association Between Mediterranean Anti-inflammatory Dietary Profile and Severity of Psoriasis: Results From the NutriNet-Santé Cohort. JAMA Dermatol..

[97] Youm Y.H., Nguyen K.Y., Grant R.W., Goldberg E.L., Bodogai M., Kim D., D’Agostino D., Planavsky N., Lupfer C., Kanneganti T.D. (2015). The ketone metabolite β-hydroxybutyrate blocks NLRP3 inflammasome-mediated inflammatory disease. Nat. Med..

[98] Castaldo G., Rastrelli L., Galdo G., Molettieri P., Rotondi Aufiero F., Cereda E. (2020). Aggressive weight-loss program with a ketogenic induction phase for the treatment of chronic plaque psoriasis: A proof-of-concept, single-arm, open-label clinical trial. Nutrition.

[99] Barrea L., Savanelli M.C., Di Somma C., Napolitano M., Megna M., Colao A., Savastano S. (2017). Vitamin D and its role in psoriasis: An overview of the dermatologist and nutritionist. Rev. Endocr. Metab. Disord..

[100] Sigmundsdottir H., Pan J., Debes G.F., Alt C., Habtezion A., Soler D., Butcher E.C. (2007). DCs metabolize sunlight-induced vitamin D3 to ‘program’ T cell attraction to the epidermal chemokine CCL27. Nat. Immunol..

[101] Patti G.J., Yanes O., Siuzdak G. (2012). Innovation: Metabolomics: The apogee of the omics trilogy. Nat. Rev. Mol. Cell Biol..

[102] Wang T.J., Larson M.G., Vasan R.S., Cheng S., Rhee E.P., McCabe E., Lewis G.D., Fox C.S., Jacques P.F., Fernandez C. (2011). Metabolite profiles and the risk of developing diabetes. Nat. Med..

[103] Yan D., Afifi L., Jeon C., Trivedi M., Chang H.W., Lee K., Liao W. (2017). The metabolomics of psoriatic disease. Psoriasis.

[104] Guo J., Zhang H., Lin W., Lu L., Su J., Chen X. (2023). Signaling pathways and targeted therapies for psoriasis. Signal Transduct. Target. Ther..

[105] Guo Q., Jin Y., Chen X., Ye X., Shen X., Lin M., Zeng C., Zhou T., Zhang J. (2024). NF-κB in biology and targeted therapy: New insights and translational implications. Signal Transduct. Target. Ther..

[106] Liu T., Zhang L., Joo D., Sun S.C. (2017). NF-κB signaling in inflammation. Signal Transduct. Target. Ther..

[107] Aparicio-Soto M., Redhu D., Sánchez-Hidalgo M., Fernández-Bolaños J.G., Alarcón-de-la-Lastra C., Worm M., Babina M. (2019). Olive-Oil-Derived Polyphenols Effectively Attenuate Inflammatory Responses of Human Keratinocytes by Interfering with the NF-κB Pathway. Mol. Nutr. Food Res..

[108] Goldminz A.M., Au S.C., Kim N., Gottlieb A.B., Lizzul P.F. (2013). NF-κB: An essential transcription factor in psoriasis. J. Dermatol. Sci..

[109] Hu X., Li J., Fu M., Zhao X., Wang W. (2021). The JAK/STAT signaling pathway: From bench to clinic. Signal Transduct. Target. Ther..

[110] Winthrop K.L. (2017). The emerging safety profile of JAK inhibitors in rheumatic disease. Nat. Rev. Rheumatol..

[111] Krueger J.G., McInnes I.B., Blauvelt A. (2022). Tyrosine kinase 2 and Janus kinase—Signal transducer and activator of transcription signaling and inhibition in plaque psoriasis. J. Am. Acad. Dermatol..

[112] Ma C., Wang Y., Dong L., Li M., Cai W. (2015). Anti-inflammatory effect of resveratrol through the suppression of NF-κB and JAK/STAT signaling pathways. Acta Biochim. Biophys. Sin..

[113] Yin Q., Wang L., Yu H., Chen D., Zhu W., Sun C. (2021). Pharmacological Effects of Polyphenol Phytochemicals on the JAK-STAT Signaling Pathway. Front. Pharmacol..

[114] Mavropoulos A., Rigopoulou E.I., Liaskos C., Bogdanos D.P., Sakkas L.I. (2013). The role of p38 MAPK in the aetiopathogenesis of psoriasis and psoriatic arthritis. Clin. Dev. Immunol..

[115] Kim E.K., Choi E.J. (2010). Pathological roles of MAPK signaling pathways in human diseases. Biochim. Biophys. Acta.

[116] Yu X.J., Li C.Y., Dai H.Y., Cai D.X., Wang K.Y., Xu Y.H., Chen L.M., Zhou C.L. (2007). Expression and localization of the activated mitogen-activated protein kinase in lesional psoriatic skin. Exp. Mol. Pathol..

[117] Mose M., Kang Z., Raaby L., Iversen L., Johansen C. (2013). TNFα- and IL-17A-mediated S100A8 expression is regulated by p38 MAPK. Exp. Dermatol..

[118] Hau C.S., Kanda N., Noda S., Tatsuta A., Kamata M., Shibata S., Asano Y., Sato S., Watanabe S., Tada Y. (2013). Visfatin enhances the production of cathelicidin antimicrobial peptide, human β-defensin-2, human β-defensin-3, and S100A7 in human keratinocytes and their orthologs in murine imiquimod-induced psoriatic skin. Am. J. Pathol..

[119] Sun Y., Zhang J., Zhai T., Li H., Li H., Huo R., Shen B., Wang B., Chen X., Li N. (2017). CCN1 promotes IL-1β production in keratinocytes by activating p38 MAPK signaling in psoriasis. Sci. Rep..

[120] Funding A.T., Johansen C., Kragballe K., Iversen L. (2007). Mitogen- and stress-activated protein kinase 2 and cyclic AMP response element binding protein are activated in lesional psoriatic epidermis. J. Investig. Dermatol..

[121] Wu Y., Liu L., Bian C., Diao Q., Nisar M.F., Jiang X., Bartsch J.W., Zhong M., Hu X., Zhong J.L. (2018). MicroRNA let-7b inhibits keratinocyte differentiation by targeting IL-6 mediated ERK signaling in psoriasis. Cell Commun. Signal. CCS.

[122] Chen J.G., Fan H.Y., Wang T., Lin L.Y., Cai T.G. (2019). Silencing KRT16 inhibits keratinocyte proliferation and VEGF secretion in psoriasis via inhibition of ERK signaling pathway. Kaohsiung J. Med. Sci..

[123] Yang J., Sun L., Han J., Zheng W., Peng W. (2019). DUSP1/MKP-1 regulates proliferation and apoptosis in keratinocytes through the ERK/Elk-1/Egr-1 signaling pathway. Life Sci..

[124] Hammouda M.B., Ford A.E., Liu Y., Zhang J.Y. (2020). The JNK Signaling Pathway in Inflammatory Skin Disorders and Cancer. Cells.

[125] Chen L., Wu J., Ren W., Yang X., Shen Z. (2013). c-Jun N-terminal kinase (JNK)-phospho-c-JUN (ser63/73) pathway is essential for FOXP3 nuclear translocation in psoriasis. J. Dermatol. Sci..

[126] Sun J., Zhao Y., Hu J. (2013). Curcumin inhibits imiquimod-induced psoriasis-like inflammation by inhibiting IL-1beta and IL-6 production in mice. PLoS ONE.

[127] Beken B., Serttas R., Yazicioglu M., Turkekul K., Erdogan S. (2020). Quercetin Improves Inflammation, Oxidative Stress, and Impaired Wound Healing in Atopic Dermatitis Model of Human Keratinocytes. Pediatr. Allergy Immunol. Pulmonol..

[128] Zhang M., Zhang X. (2019). The role of PI3K/AKT/FOXO signaling in psoriasis. Arch. Dermatol. Res..

[129] Teng Y., Fan Y., Ma J., Lu W., Liu N., Chen Y., Pan W., Tao X. (2021). The PI3K/Akt Pathway: Emerging Roles in Skin Homeostasis and a Group of Non-Malignant Skin Disorders. Cells.

[130] Liu Y., Luo W., Chen S. (2011). Comparison of gene expression profiles reveals aberrant expression of FOXO1, Aurora A/B and EZH2 in lesional psoriatic skins. Mol. Biol. Rep..

[131] Mitra A., Raychaudhuri S.K., Raychaudhuri S.P. (2012). IL-22 induced cell proliferation is regulated by PI3K/Akt/mTOR signaling cascade. Cytokine.

[132] Huang T., Lin X., Meng X., Lin M. (2014). Phosphoinositide-3 kinase/protein kinase-B/mammalian target of rapamycin pathway in psoriasis pathogenesis. A potential therapeutic target?. Acta Derm.-Venereol..

[133] Patel A.B., Tsilioni I., Weng Z., Theoharides T.C. (2018). TNF stimulates IL-6, CXCL8 and VEGF secretion from human keratinocytes via activation of mTOR, inhibited by tetramethoxyluteolin. Exp. Dermatol..

[134] Chen L., Wu J., Pier E., Zhao Y., Shen Z. (2013). mTORC2-PKBα/Akt1 Serine 473 phosphorylation axis is essential for regulation of FOXP3 Stability by chemokine CCL3 in psoriasis. J. Investig. Dermatol..

[135] Akinduro O., Sully K., Patel A., Robinson D.J., Chikh A., McPhail G., Braun K.M., Philpott M.P., Harwood C.A., Byrne C. (2016). Constitutive Autophagy and Nucleophagy during Epidermal Differentiation. J. Investig. Dermatol..

[136] Roy T., Boateng S.T., Uddin M.B., Banang-Mbeumi S., Yadav R.K., Bock C.R., Folahan J.T., Siwe-Noundou X., Walker A.L., King J.A. (2023). The PI3K-Akt-mTOR and Associated Signaling Pathways as Molecular Drivers of Immune-Mediated Inflammatory Skin Diseases: Update on Therapeutic Strategy Using Natural and Synthetic Compounds. Cells.

[137] Albanesi C., Madonna S., Gisondi P., Girolomoni G. (2018). The Interplay Between Keratinocytes and Immune Cells in the Pathogenesis of Psoriasis. Front. Immunol..

[138] Griffiths C.E.M., Armstrong A.W., Gudjonsson J.E., Barker J. (2021). Psoriasis. Lancet.

[139] Li H., Yao Q., Mariscal A.G., Wu X., Hülse J., Pedersen E., Helin K., Waisman A., Vinkel C., Thomsen S.F. (2018). Epigenetic control of IL-23 expression in keratinocytes is important for chronic skin inflammation. Nat. Commun..

[140] Brembilla N.C., Senra L., Boehncke W.H. (2018). The IL-17 Family of Cytokines in Psoriasis: IL-17A and Beyond. Front. Immunol..

[141] Huangfu L., Li R., Huang Y., Wang S. (2023). The IL-17 family in diseases: From bench to bedside. Signal Transduct. Target. Ther..

[142] Liu T., Li S., Ying S., Tang S., Ding Y., Li Y., Qiao J., Fang H. (2020). The IL-23/IL-17 Pathway in Inflammatory Skin Diseases: From Bench to Bedside. Front. Immunol..

[143] Palazzo E., Lotti R., Quadri M., Pincelli C., Marconi A. (2025). IL-17 Ligand and Receptor Family Members Are Differentially Expressed by Keratinocyte Subpopulations and Modulate Their Differentiation and Inflammatory Phenotype. Int. J. Mol. Sci..

[144] Hao J.Q. (2014). Targeting interleukin-22 in psoriasis. Inflammation.

[145] Bukvić Mokos Z., Tomić Krsnik L., Harak K., Marojević Tomić D., Tešanović Perković D., Vukojević M. (2025). Vitamin D in the Prevention and Treatment of Inflammatory Skin Diseases. Int. J. Mol. Sci..

[146] Lowes M.A., Chamian F., Abello M.V., Fuentes-Duculan J., Lin S.L., Nussbaum R., Novitskaya I., Carbonaro H., Cardinale I., Kikuchi T. (2005). Increase in TNF-alpha and inducible nitric oxide synthase-expressing dendritic cells in psoriasis and reduction with efalizumab (anti-CD11a). Proc. Natl. Acad. Sci. USA.

[147] Papayannopoulos V., Metzler K.D., Hakkim A., Zychlinsky A. (2010). Neutrophil elastase and myeloperoxidase regulate the formation of neutrophil extracellular traps. J. Cell Biol..

[148] Río C.D., Millán E., García V., Appendino G., DeMesa J., Muñoz E. (2018). The endocannabinoid system of the skin. A potential approach for the treatment of skin disorders. Biochem. Pharmacol..

[149] Wroński A., Wójcik P. (2022). Impact of ROS-Dependent Lipid Metabolism on Psoriasis Pathophysiology. Int. J. Mol. Sci..

[150] Gu X., Cai Z., Cai M., Liu K., Liu D., Zhang Q., Tan J., Ma Q. (2018). AMPK/SIRT1/p38 MAPK signaling pathway regulates alcohol-induced neurodegeneration by resveratrol. Mol. Med. Rep..

[151] Ruderman N.B., Xu X.J., Nelson L., Cacicedo J.M., Saha A.K., Lan F., Ido Y. (2010). AMPK and SIRT1: A long-standing partnership?. Am. J. Physiol. Endocrinol. Metab..

[152] Słuczanowska-Głabowska S., Salmanowicz M., Staniszewska M., Pawlik A. (2023). The Role of Sirtuins in the Pathogenesis of Psoriasis. Int. J. Mol. Sci..

[153] Kauppinen A., Suuronen T., Ojala J., Kaarniranta K., Salminen A. (2013). Antagonistic crosstalk between NF-κB and SIRT1 in the regulation of inflammation and metabolic disorders. Cell Signal.

[154] Wang Z.H., Zhan-Sheng H. (2018). Catalpol inhibits migration and induces apoptosis in gastric cancer cells and in athymic nude mice. Biomed. Pharmacother..

[155] Xie S., Su Z., Zhang B., Ge J., Song S., Sun G., Sun X., Yi L., Wang Y., Sun W. (2015). SIRT1 Activation Ameliorates Aldara-Induced Psoriasiform Phenotype and Histology in Mice. J. Investig. Dermatol..

[156] Yang H., Zhang W., Pan H., Feldser H.G., Lainez E., Miller C., Leung S., Zhong Z., Zhao H., Sweitzer S. (2012). SIRT1 activators suppress inflammatory responses through promotion of p65 deacetylation and inhibition of NF-κB activity. PLoS ONE.

